# *Arabidopsis* thimet oligopeptidases are redox-sensitive enzymes active in the local and systemic plant immune response

**DOI:** 10.1016/j.jbc.2021.100695

**Published:** 2021-04-22

**Authors:** Thualfeqar Al-Mohanna, Najmeh Nejat, Anthony A. Iannetta, Leslie M. Hicks, George V. Popescu, Sorina C. Popescu

**Affiliations:** 1 Department of Biochemistry, Molecular Biology, Entomology, and Plant Pathology, Mississippi State University, Mississippi State, Mississippi, USA; 2 Department of Chemistry, The University of North Carolina at Chapel Hill, Chapel Hill, North Carolina, USA; 3 Institute for Genomics, Biocomputing, and Biotechnology, Mississippi State University, Mississippi State, Mississippi, USA

**Keywords:** thimet oligopeptidase, *Arabidopsis*, disulfide, oxidative activation, redox-sensitive thiol, ESI-MS, electrospray ionization–mass spectrometry, ETI, effector-triggered immunity, GSH, glutathione, NTRC, NADPH-dependent thioredoxin reductase C, OOP, organellar oligopeptidase, ROS, reactive oxygen species, SAR, systemic acquired response, SEC, size-exclusion chromatography, TOP, thimet oligopeptidase

## Abstract

Upon pathogen infection, receptors in plants will activate a localized immune response, the effector-triggered immunity (ETI), and a systemic immune response, the systemic acquired response (SAR). Infection also induces oscillations in the redox environment of plant cells, triggering response mechanisms involving sensitive cysteine residues that subsequently alter protein function. *Arabidopsis thaliana* thimet oligopeptidases *TOP1* and *TOP2* are required for plant defense against pathogens and the oxidative stress response. Herein, we evaluated the biochemical attributes of TOP isoforms to determine their redox sensitivity using *ex vivo Escherichia coli* cultures and recombinant proteins. Moreover, we explored the link between their redox regulation and plant immunity in wild-type and mutant *Arabidopsis* lines. These analyses revealed that redox regulation of TOPs occurs through two mechanisms: (1) oxidative dimerization of full-length TOP1 *via* intermolecular disulfides engaging cysteines in the N-terminal signal peptide, and (2) oxidative activation of all TOPs *via* cysteines that are unique and conserved. Further, we detected increased TOP activity in wild-type plants undergoing ETI or SAR following inoculation with *Pseudomonas syringae* strains. Mutants unable to express the chloroplast NADPH-dependent thioredoxin reductase C (NTRC) showed elevated TOP activity under unstressed conditions and were SAR-incompetent. A *top1top2* knockout mutant challenged with *P. syringae* exhibited misregulation of ROS-induced gene expression in pathogen-inoculated and distal tissues. Furthermore, TOP1 and TOP2 could cleave a peptide derived from the immune component ROC1 with distinct efficiencies at common and specific sites. We propose that *Arabidopsis* TOPs are thiol-regulated peptidases active in redox-mediated signaling of local and systemic immunity.

Hindering pathogen proliferation and tissue damage is a common challenge for organisms across the tree of life. Plants respond to pathogen infection by activating localized and systemic defense responses. Intracellular immune receptors, such as RPS2, initiate the effector-triggered immune response (ETI) by recognizing effectors such as *Pseudomonas syringae*'s AvrRpt2 at the site of pathogen infection (1). Following this localized immune response, organs distal from the infection site activate the systemic acquired resistance (SAR) (2), a type of systemic immunity that confers long-lasting protection to secondary infections (3).

As a central element in the plant interaction with pathogens, the redox state in the plant cells is dynamic and tightly controlled (4). The immune response activation relies on the rapid accumulation of reactive oxygen species (ROS) in multiple subcellular compartments. Oxidants such as hydrogen peroxide (H_2_O_2_) are generated metabolically and enzymatically by, for example, NADPH-dependent oxidases and may accumulate within precise spatial and temporal parameters during an immune response (5). The sudden ROS upsurge postinfection, called the “oxidative burst,” causes redox modifications in proteins required to activate local and systemic immunity and the programmed cell death pathways (6, 7). Protein thiols toggling between reduced and oxidized states constitute a universal redox-sensing mechanism that modulates host signaling in response to cellular redox potential (8) and critical for pathogenesis (9). Coordination among diverse subcellular compartments, including the cytosol, chloroplast, and mitochondria, has been recognized as essential for cellular redox homeostasis. Reductive systems, including glutathione (GSH), NAD/NADP, and ascorbate, function across multiple cellular compartments and constitute an essential conduit for ROS processing and redox signaling (10).

Although immune-associated redox processes have been studied since the 1970s (11, 12), and numerous studies since have demonstrated their essentiality to immune signal transduction (10, 11, 12, 13, 14, 15), our knowledge of the redox biology underlying plant immunity is still basic (16, 17, 18). Redox proteomics has provided evidence of hundreds of redox-sensitive protein thiols; however, few are associated with a specific function (19, 20). One well-studied redox-modulated immune component is the transcription cofactor NPR1, which is converted by thioredoxins from an inactive oligomer to active monomers to induce SAR *via* a pathway controlled by the plant defense hormone salicylic acid (SA) (21).

Recent studies highlight the interconnectivity between cytosolic and organellar protein homeostasis networks in metazoan's response to oxidative stress (22, 23). Our understanding of the plant proteostasis networks and their roles in immunity and redox signaling stress is emerging (24, 25). In this context, we study thimet oligopeptidases (TOPs), a family of evolutionarily conserved peptidases with a metal-binding His-Glu-Xaa-Xaa-His (HEXXH) active site motif (26). Plant TOPs are encoded by multiple genes translated into proteins with medium-to-low similarity to metazoan TOPs (27, 28). *Arabidopsis thaliana* TOP1, or organellar oligopeptidase (OOP), contains an N-terminal dual-localization signal (transit) peptide of ∼100 amino acids for transport into chloroplasts and mitochondria (28, 29, 30). Proteomic studies have also detected cytosolic full-length TOP1 (31). Organellar TOP1 is a component of the protein import pathways with roles in processing transit peptides (29). The closely related cytosolic TOP2 (CyOOP) was associated with the degradation of oxidized peptide products released from the 20S proteasome (32). TOP1 lacking the predicted target sequence (herein named ^ΔSP^TOP1) was crystallized in a closed conformation resembling the *Escherichia coli* dipeptidyl carboxypeptidase (29). TOP2 was crystallized in an open conformation resembling metazoan THOPs (33).

We previously demonstrated that TOP1 and TOP2 are components of plant immune pathways necessary to activate ETI and sustain an optimal oxidative burst. We showed that SA bound and inhibited TOP1 activity and facilitated its dimerization, whereas SA interacted with ^ΔSP^TOP1 and TOP2 with much lower affinity and had a lesser impact on their activity (28, 34). A *top1top2* knockout mutant had increased susceptibility to pathogen infection, abnormal oxidative burst, and defective levels of proteome oxidation during ETI (25, 28). Biochemical studies of TOPs suggested a possible redox regulation of their functions (34). These studies collectively confirmed the cooperative roles of *A. thaliana TOPs* in immunity and oxidative stress and highlighted apparent differences in TOPs biochemical characteristics. Here, we undertake a detailed comparative investigation of the redox sensitivity of *A. thaliana* TOPs. We address the roles of TOPs cysteine residues for TOPs self-interaction and enzymatic activity *ex vivo* and *in vitro* and the identity of redox-sensitive cysteines. Further, we assess TOPs activity in the context of plant immune response, evaluate an oxidoreductase's role as a TOPs regulator, and test TOPs cleavage specificity on a peptide derived from a critical immune regulator.

## Results

### Structural characteristics of TOP1 and TOP2 influence their physicochemical properties

**Figure 1:**
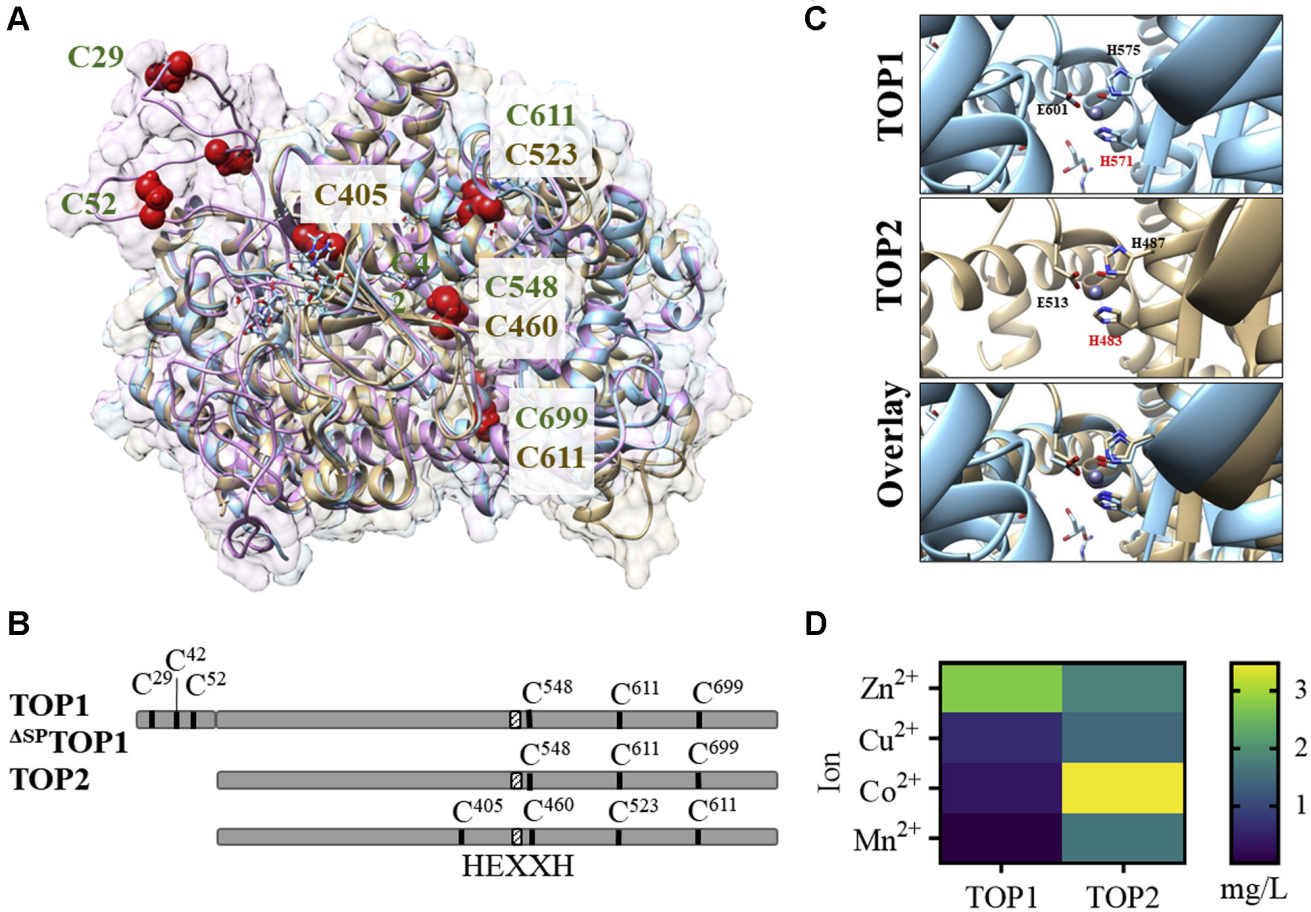
**Biochemical and structural characteristics of plant TOPs.***A*, superimposed three-dimensional structures of full-length TOP1 (*magenta*), TOP1 lacking the signal peptide (^ΔSP^TOP1, *blue*), and TOP2 (*brown*). *B*, diagram showing cysteine residues and the position of the conserved metallopeptidase active site motif HEXXH in plant TOPs. *C*, a Chimera-derived 3D model of the metallopeptidase conserved site, HEXXH in TOP1, TOP2, and a superimposed view. In TOP1, one Zn^2+^ is tetrahedrally coordinated by the active site residues H^571^, H^575^, and in TOP2, Zn^2+^ is coordinated by the active site residues H^483^, H^487^, and E^513^. *D*, ion-binding properties of TOP1 and TOP2 were measured by inductively coupled plasma mass spectrometry.

We hypothesized that the structural characteristics of *A. thaliana* TOPs determine their biochemical properties. To gain insight into potential differences among TOP isoforms, we generated a three-dimensional (3D) model of full-length TOP1 *via* homology modeling (35) using as a template the structure of the organellar TOP1 lacking the N-terminal signal peptide sequence (29). We compared the TOP1 model with ^ΔSP^TOP1 (29) and TOP2 (33) structures to assess the location and conservation of Cys residues (Fig. 1*A*). Three Cys residues situated in the peptidase domain are conserved – C^548/460^, C^611/523^, and C^699/611^ in TOP1/TOP2. C^29^, C^42^, and C^52^ are located in the signal peptide of TOP1 and thus unique for the full-length TOP1, whereas C^405^ is unique for TOP2 (Fig. 1*B*). Measurement of Cys-to-Cys distances in ^ΔSP^TOP1, TOP2, and the modeled TOP1 showed that Cys residues are positioned within distances larger than ∼2 Å required for forming intramolecular disulfide bonds (36). Notably, C^29^ and C^52^ in TOP1's signal peptide are predicted to be solvent-exposed, whereas C^405^ of TOP2 has the lowest buried coefficient among its cysteines, suggesting a potential for the redox regulation of these solvent-exposed residues (Fig. S1).

A characteristic feature of TOPs is the hexapeptide zinc-binding motif (HEXXGH). In the plant TOPs, as in other zinc metallopeptidases, His, His, and Glu residues coordinate with Zn^2+^ to form hydrogen bonds with acidic amino acid residues (Glu or Asp) (Fig. 1*C*). The binding site's geometry in many bacterial metalloproteases allows for Zn^2+^ substitution, with subsequent effects in some cases, on other properties such as protein stability (37, 38). To determine the metal ion-binding properties of TOPs, recombinant TOP1-His and TOP2-His were purified from *E. coli* cultures. The 6xHis tags were removed by thrombin cleavage before the incubation of TOP1 and TOP2 with metal ions and analysis by inductively coupled mass spectrometry. We found that TOP1 preferentially bound Zn^2+^ (2.7 mg/L) and had lower affinities for Cu^2+^, Co^2+^ (∼0.5 mg/L), and Mn^2+^ (<0.01 mg/L) (Fig. 1*D*). Comparatively, TOP2 had a more permissive ion-binding ability; TOP2 bound Co^2+^ (3.4 mg/L), Zn^2+^, Mn^2+^, and Cu^2+^ (each at ∼1.5–2 mg/L) (Fig. 1*D*). Altogether, differences in TOPs primary and tertiary structures suggest distinct modes of regulation.

### Oxidation promotes the dimerization of TOP1 but not of TOP2

**Figure 2:**
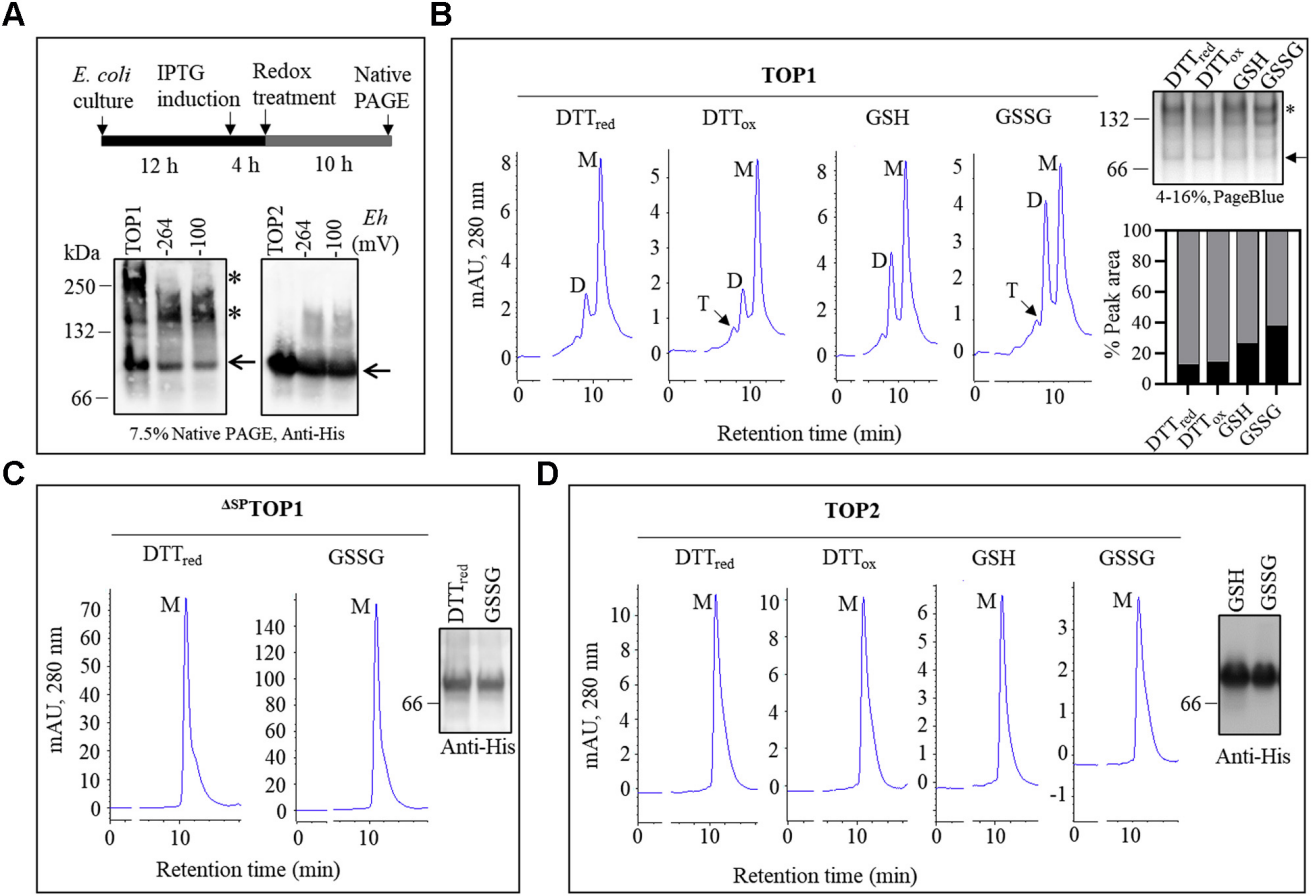
**TOP1 oxidation promotes its self-oligomerization.***A*, analysis of TOP1 and TOP2 self-interaction behavior *ex vivo*, showing the diagram of the experimental strategy and anti-His immunoblots of total protein extracts from *TOP1* and *TOP2*-expressing cultures subjected to redox treatments (GSH, –264 mV and GSSG, –100 mV), and purified proteins as controls. *Arrows* show the position of monomers, and *asterisks* (∗) point to the higher molecular bands. MW markers and redox potentials (*Eh*) are shown. *B–D*, size-exclusion chromatography (SEC) comparing elution profiles of recombinant purified TOP1 (*B*), ^ΔSP^TOP1 (*C*), and TOP2 (*D*) following incubation with reducing or oxidative agents (DTT_red_, DTT_ox,_ GSH, and GSSG). *B*, a native PAGE of purified and treated TOP1 samples stained with PageBlue shows monomeric and high-MW TOP1 isoforms (*upper right*), and a graph with bars showing peak area quantification with percentages of monomers (M) and dimers (D) (*lower right*). *C* and *D*, anti-His immunoblots were done for purified and treated ^ΔSP^TOP1 and TOP2.

We hypothesized that the observed structural differences between TOP1 and TOP2 impact redox behaviors such as self-interaction. We first tested this hypothesis using an *ex vivo* redox titration system adapted from (39). *E. coli* cultures expressing recombinant TOPs were treated with defined ratios of GSH/GSSG and DTT_red_/DTT_ox_ solutions to yield a range of reducing and oxidizing environments (–360 to –100 mV) (Fig. 2*A* and Supplemental Methods). Ten hours posttreatment (hpt), total protein extracts were separated on native PAGE and subjected to immunoblotting using anti-His antibodies. Following GSH/GSSG treatments, the anti-His antibody detected monomeric TOP1 and higher-MW bands in total extracts and a control purified TOP1 sample (Fig. 2*A*, left). The monomeric and high-MW bands were also detected in extracts from cells subjected to the full range of redox treatments with GSH/GSSG and DTT_red_/DTT_ox_ (Fig. S2*A*).

A similar analysis of *TOP2*-expressing cells treated with GSH or GSSG detected solely monomeric TOP2 in total extracts and a purified TOP2 sample (Fig. 2*A*, right). Notably, in extracts run on a 3–12% native PAGE, the anti-His antibody detected a slower-migrating TOP2 band (Fig. S2*B*), which may be dimeric TOP2. We tested this possibility by analyzing total extracts from DTT_red_- and GSSG-treated *TOP2*-expressing cells using 2D electrophoresis; only TOP2 monomers were detected under both conditions (Fig. S2*D*).

The self-interaction behavior of TOPs under diverse redox conditions was further analyzed using purified recombinant proteins and size-exclusion HPLC (SEC). Protein size markers, including bovine serum albumin (BSA) known to dimerize (40), were run through the size-exclusion chromatography (SEC) column, and a regression line was plotted using the retention times (Rt) and MWs (Fig. S2*C*). Purified recombinant TOP1, ^ΔSP^TOP1, and TOP2 were treated with DTT_red_, DTT_ox_, GSG, or GSSG and run through the SEC column. We found that TOP1 eluted mainly as two peaks, one prominent monomeric peak and one smaller corresponding to dimeric TOP1, alongside a low-abundance trimeric peak (Fig 2*B* and Fig. S2*C*). A quantitative assessment of peak area showed that the TOP1 M/D ratio remained constant post-DTT_red_ and -DTT_ox_ incubations (∼7/1 under both conditions); however, the M/D ratio decreased markedly postglutathione treatments (3/1 and 2/1 post-GSH and -GSSG, respectively) (Fig. 2*B*, lower right inset). TOP1 monomeric and higher-MW structures were also visible in the SEC-analyzed TOP1 samples separated on native PAGE and stained with a generic protein stain (Fig. 2*B*, upper right inset). In contrast to the full-length isoform, ^ΔSP^TOP1 was detected solely as a monomer following reductive (DTT_red_) and oxidative (GSSG) treatments, as shown by SEC analysis (Fig. 2*C*) and anti-His immunoblotting (Fig. 2*C*, right inset). TOP2 analysis revealed that incubation with DTT_red_, DTT_ox_, GSH, or GSSG failed to induce its dimerization; solely monomers were detected by SEC analysis (Fig. 2*D*) and an anti-His immunoblot of the SEC-analyzed samples (Fig. 2*D*, inset).

### Mapping cysteine residues involved in TOP1 intermolecular cross-linking

So far, the results indicated that TOPs exist predominantly as monomers *ex vivo* and *in vitro* and brought forth the prospect that the signal peptide of TOP1 is required for its dimerization. We used site-directed mutagenesis to generate mutant TOPs with individual cysteines changed to redox-insensitive alanine. Recombinant proteins were expressed in *E. coli* (Fig. S3*A*), purified, and treated with oxidizing agents prior to SEC analysis; untreated purified proteins served as controls.

**Figure 3:**
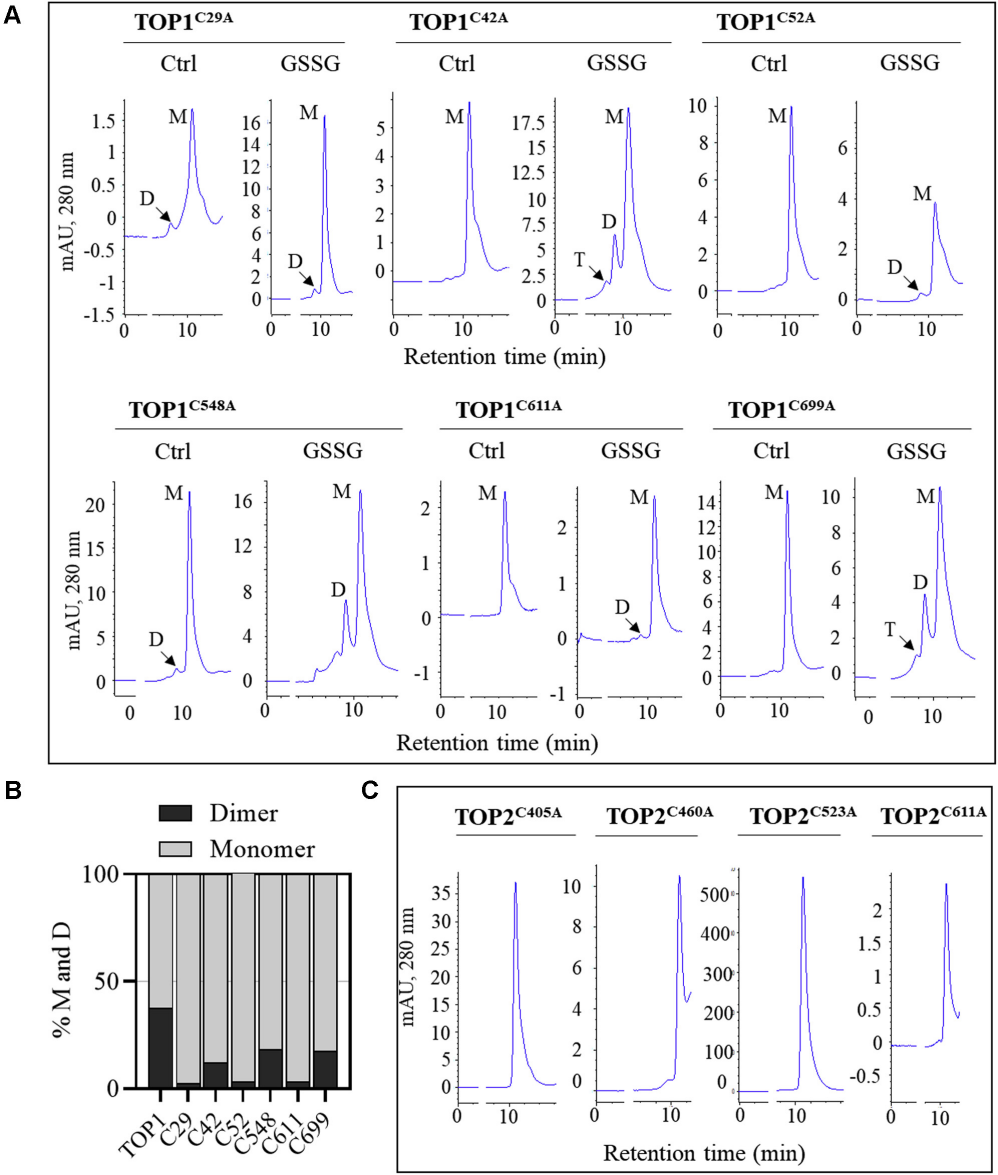
**Redox-sensitive cysteine residues in TOP1 signal peptide and peptidase domain control TOP1 homodimerization.***A*, size-exclusion chromatography (SEC) analysis of purified TOP1 Cys-to-Ala mutants in the absence of redox treatments (Ctrl) and after incubation with GSSG. The chromatograms show the position of monomeric (M), dimeric (D), and trimeric (T) peaks (n = 2). *B*, peak area quantification for TOP1 samples analyzed in A showing percentage of TOP1 dimers in one representative experiment. *C*, SEC analysis of purified TOP2 Cys-to-Ala mutants after incubation with GSSG (n = 2).

Following GSSG treatment, TOP1^C29A^, TOP1^C52A^, and TOP1^C611A^ showed smaller dimeric peaks than TOP^C42A^, TOP^C548A^, or TOP1^C699A^ (Fig. 3*A*). Moreover, unlike the native TOP1, untreated TOP1^C52A^, TOP1^C611A^, and TOP^C42A^ mutants eluted solely as monomers. TOP1^C29A^ consistently showed low peak intensities in SEC, suggesting a more general effect of this mutation on the monomer stability. A quantitative assessment of the peaks showed that C^52^ and C^611^ had the highest impact on TOP1 dimerization (Fig. 3*B*). *Ex vivo* analyses detected TOP1 in high-MW complexes (Fig. 2*A*); therefore, we tested if Cys-to-Ala mutations impaired TOP1 ability to form these structures in *E. coli* cells treated with GSH or GSSG. Total extracts were separated in nonreducing PAGE, and TOP1 was visualized using the anti-His antibody. Consistent with the *in vitro* analysis, mutagenesis of residues in TOP1 signal peptide markedly reduced the accumulation of high-MW complexes compared with the native TOP1 (Fig. S3*B*).

SEC analysis of the GSSG-treated TOP2 mutants – C^405^A, C^460^A, C^523^A, and C^611^A – determined that none of the individual mutations affected TOP2 monomerization, as shown by the elution of all mutant proteins in monomer-size peaks (Fig. 3*C*). TOP2 mutant proteins were also analyzed *ex vivo* using *E. coli* cultures expressing individual mutants treated with GSH or GSSG. The anti-His antibody detected solely monomeric TOP2 in the respective total extracts; the monomeric signal intensity was weaker in mutants than the native TOP2, suggesting that these mutations may impact monomer accumulation or stability (Fig. S3*C*).

**Figure 4:**
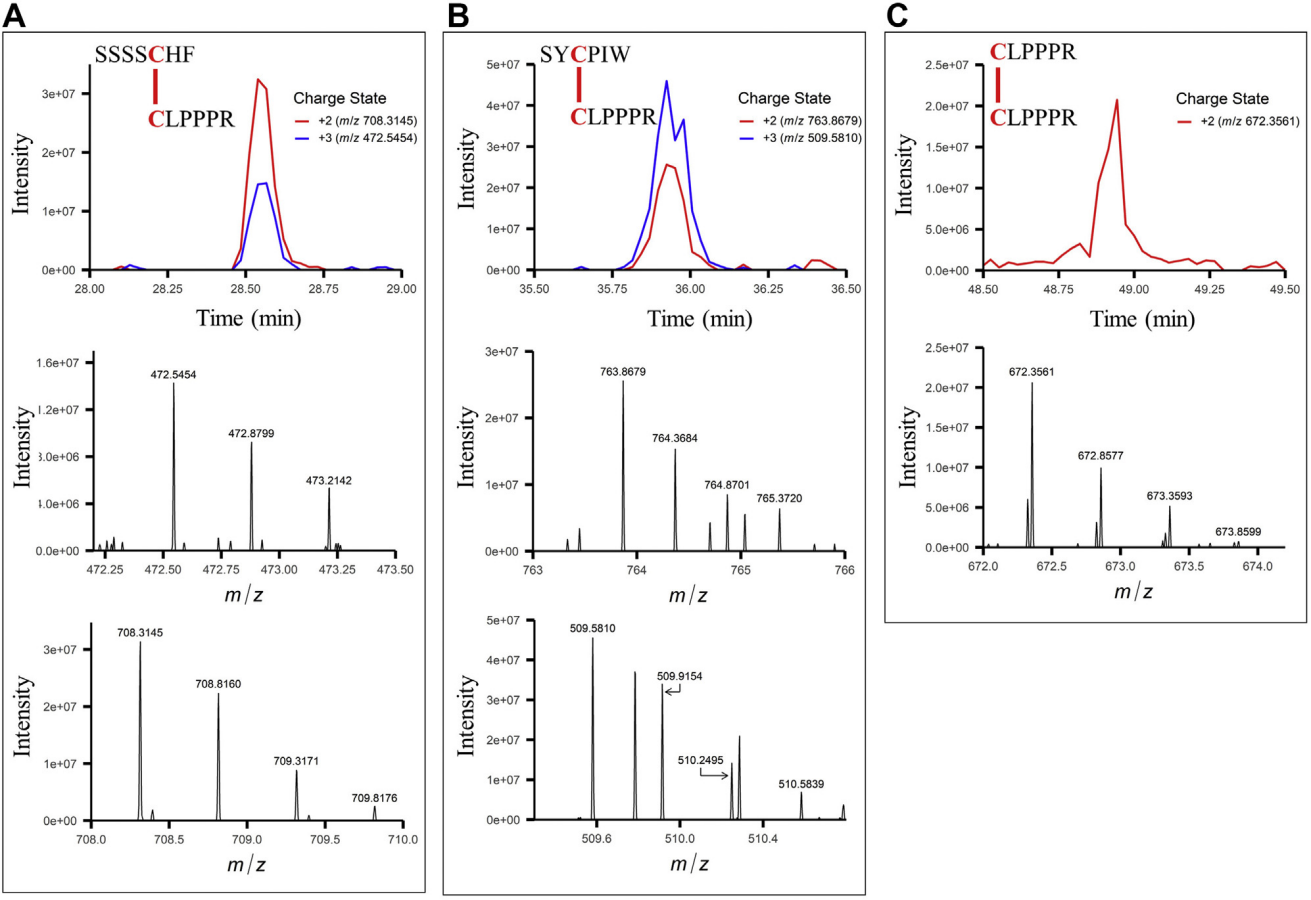
**Disulfide-bound cysteines involved in TOP1 oligomerization.***A*, extracted ion chromatograms and mass spectra at 28.54 min zoomed into the isotopic distributions for two charge states (*m/z* = 708.3145, +2 charge state, and *m/z* = 472.5454, +3 charge state) of SSSSCHF disulfide bound to CLPPPR corresponding to TOP1 C^29^ and C^52^. *B*, extracted ion chromatograms and mass spectra at 35.92 min zoomed into the isotopic distributions for two charge states (*m/z* = 763.8679, +2 charge state, and *m/z* = 509.5810, +3 charge state) of SYCPIW disulfide bound to CLPPPR corresponding to TOP1 C^42^ and C^52^. *C*, extracted ion chromatogram and mass spectrum at 48.94 min zoomed into the isotopic distribution for one charge state (*m/z* = 672.3561, +2 charge state) of CLPPPR disulfide bound to CLPPPR corresponding to TOP1 C^52^ and C^52^. All observed masses match with the theoretical peptide masses within 2 ppm mass error.

Exposure to oxidizing conditions might facilitate TOP1 dimerization *via* disulfides. We performed LC-MS to identify cysteines involved in TOP1 dimerization. Native PAGE bands from TOP1-expressing *E. coli* cells treated with GSSG were subjected to in-gel digestion with trypsin and chymotrypsin and analyzed. Reduction and alkylation of cysteines, typically employed in bottom-up proteomics, were omitted to preserve disulfide bonds. A protein database search was used first to confirm TOP1 detection (Fig. S4). The LC-MS data were manually interrogated for ions corresponding to the charge states of theoretical, disulfide-bound dipeptides. This strategy revealed three disulfide-bound dipeptides, showing intermolecular connectivity between C^29^–C^52^ (Fig. 4*A*), C^42^–C^52^ (Fig. 4*B*), and C^52^–C^52^ (Fig. 4*C*). These matched theoretical dipeptide masses with <2 ppm mass error. These dipeptides were not detected in similarly processed monomeric TOP1 samples, providing further confidence in these peptide assignments.

### Redox-sensitive cysteine residues control TOP1 and TOP2 catalytic activity

**Figure 5:**
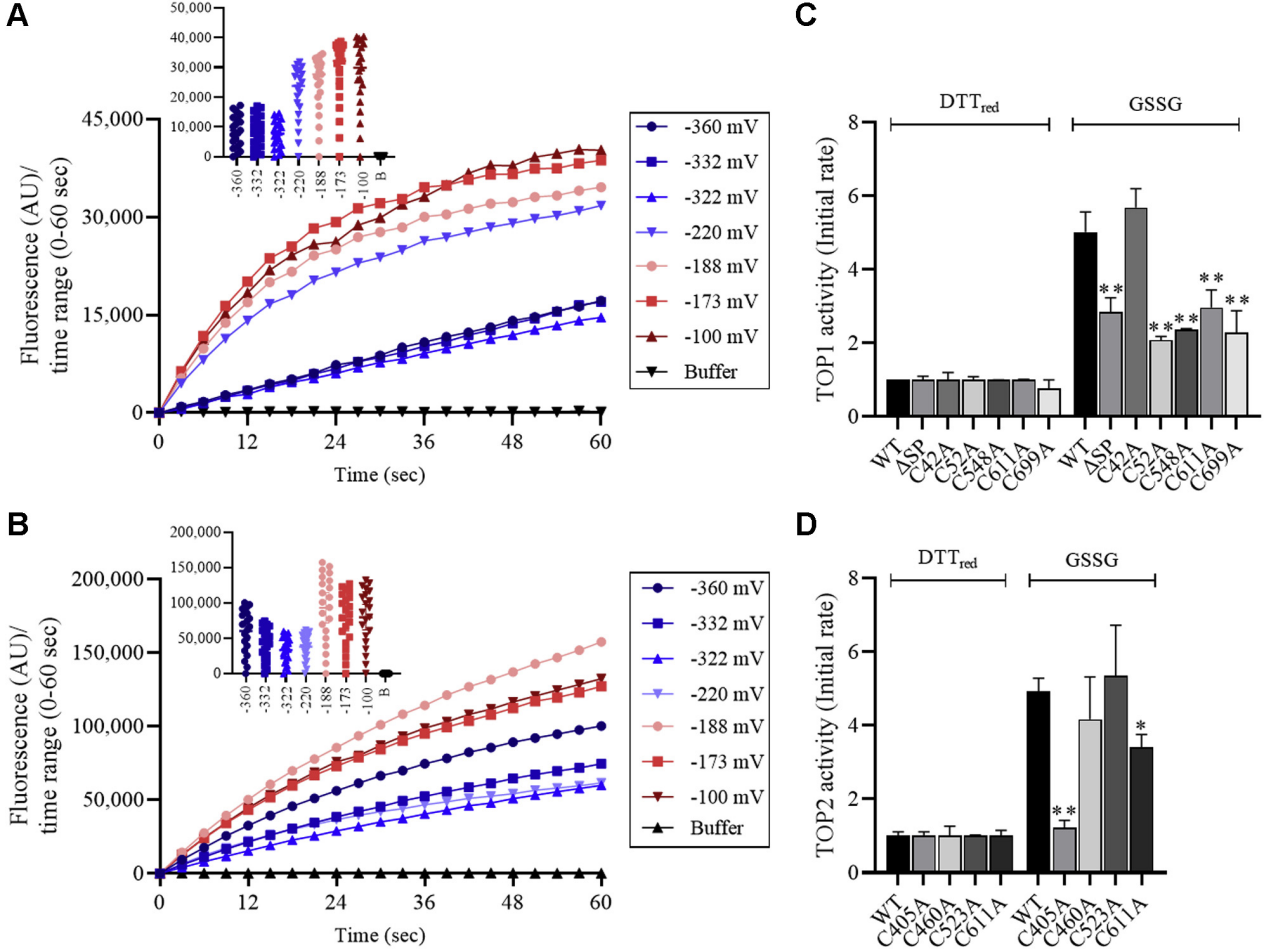
**Oxidative activation of TOPs.***A* and *B*, purified recombinant TOP1 (*A*) and TOP2 (*B*) were incubated in the presence of oxidized and reduced reagents at defined thiol/disulfide ratios. Fluorescence emitted by the cleaved substrate in the first 60 s (sec) of the reaction was plotted against time and the values of ambient redox environment potential (Eh, mV) in the inset graphs. *C* and *D*, purified recombinant wild-type TOP1 (*C*) and TOP2 (*D*) proteins were incubated for 90 min in the presence of DTT_red_ or GSSG. TOPs activity was assayed on the fluorogenic peptide substrate at λ_ex_ = 330 nm and λ_em_ = 400 nm, over 100 s. Initial rates were measured from data shown in A and B and values from DTT_red_ reactions used to for normalization. Bars represent initial reaction rates with standard deviation over independent experiments (n ≥ 3). *p* Values <0.05 (∗); <0.01 (∗∗) calculated using ordinary one-way ANOVA.

Changes in the redox state of protein thiols may impact TOPs activity level. To determine the effect of the ambient redox potential on TOP activity, purified TOP1 and TOP2 were incubated in solutions containing defined ratios of DTT_red_/DTT_ox_ and GSH/GSSG. Treated TOPs were mixed with the TOP-specific fluorogenic peptide (Mca-PLGPK(DNP)-OH) (41). We found that oxidation markedly augmented TOP1 activity, assessed by measuring the cleaved product's fluorescence (Fig. 5*A*). TOP1 activity was maintained at a low and constant level for the –360 to –322 mV range but gradually increased in the –220 to –100 mV range. The midpoint redox potential (E_m_) was estimated from the nonlinear best fit of the fraction of reduced protein *versus* the ambient redox potentials (42, 43). We estimated TOP1's E_m_ at –199.5 ± 1.4 mV mV at pH 7.0 (95% confidence interval, –203 to –195.9 mV) when fitting the Nernst equation for a single, two-electron redox reaction (Fig. S5). Similar to TOP1, TOP2 activity was also increased by oxidation; on average, TOP2 was 3-fold more active post-GSH/GSSG compared with DTT_red_/DTT_ox_ treatments; unlike TOP1, TOP2 activity gradually decreased with increasing oxidation within the E_m_ range of both types of treatments, although it retained over 85% activity at strongly oxidizing E_m_ values (Fig. 5*B*). Overall, the results indicate that oxidation augments TOPs activity.

Next, to determine the cysteine residues necessary for TOPs oxidative activation, we compared the oxidized (GSSG-treated) and reduced (DTT_red_-treated) mutants and native TOPs. GSSG treatment augmented TOP1 native and mutant proteins. However, we measured significantly lower activity levels for ^ΔSP^TOP1 and four of the Cys-to-Ala mutants (C^52^A, C^548^A, C^611^A, and C^699^A), suggesting that multiple cysteines contribute to TOP1 oxidative activation (Fig. 5*C*). Measurements of native and mutant TOP2 proteins verified the activity enhancement of the oxidized native TOP2 and revealed that two mutations had a significant impact: the C^405^A mutation abolished, and C^611^A impaired TOP2 oxidative activation (Fig. 5*D*).

### TOP activity increases during the plant immune response and is under the regulation of the NTRC oxidoreductase

**Figure 6:**
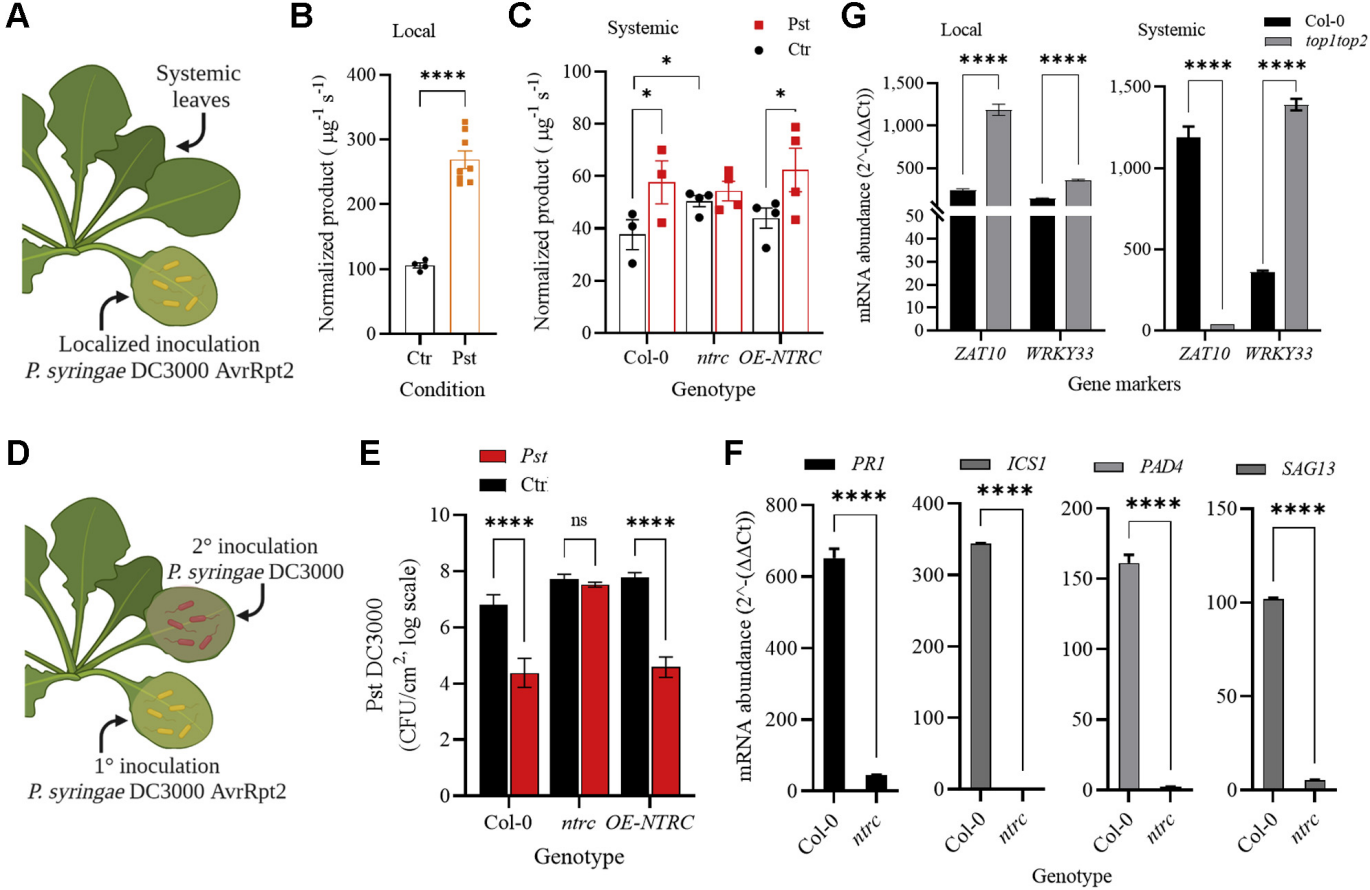
**Activation of the local and systemic immunity enhances TOPs enzymatic activity.***A*, activation of ETI in *A. thaliana* leaves. *B*, TOP activity in wild type Col-0 infiltrated with *P. syringae* AvrRpt or MgCl_2_ buffer as a control (Ctr). *C*, TOP specific activity measured in Col-0, *ntrc*, and *OE-NTRC* total protein extracts obtained from systemic leaves collected from plants locally challenged with *P. syringae* AvrRpt or controls (Ctr) infiltrated with MgCl_2_. *D*, activation of SAR in *A. thaliana* leaves. *B* and *C*, bars represent the accumulation of reaction product for each genotype and condition with the standard deviation, n = 4; enzyme activities were measured at λ_ex_ = 330 nm and λ_em_ = 400 nm, for 100 s. *E*, quantification of *P. syringae* DC3000 growth (Colony-forming units, Cfu/cm^2^) in plants treated as shown in (*D*) at 2 days postinoculation (dpi); n = 4. *F*, quantification of *PR1*, *ICS1*, *PAD4*, and *SAG13* transcripts in systemic tissue of Col-0 and *ntrc* plants at 48 hpi post-PstAvrRpt inoculation. *G*, quantification of ROS-inducible gene transcripts in local and systemic tissues at 48 hpi with PstAvrRpt. *F* and *G*, fold-change gene expression (2^ˆ^−(ΔΔCt)) was obtained from raw Ct values normalized to a ubiquitin housekeeping gene and buffer-infiltrated controls. *p* Values <0.0001 (∗∗∗∗); <0.05 (∗) show significance calculated using multiple comparisons, two-way ANOVA; ns: not significant.

Our observations herein pointed to oxidative activation as a common characteristic of plant TOPs. We previously demonstrated that *TOP1* and *TOP2* are required for the optimal activation of the oxidative burst and ETI triggered by infection with *P. syringae* DC3000 AvrRpt2 (PstAvrRpt2) (44, 45). Thus, TOP enzymatic activities might be required and influenced by the cellular redox homeostasis fluctuations characteristic of the plant immune response. We determined TOP activity in wild-type Col-0 plants undergoing PstAvrRpt2-triggered ETI and compared them to buffer-infiltrated controls (Fig. 6*A*). TOP activity on the fluorogenic TOP substrate was measured in total protein extracts (100 μg) from leaves collected 48 h postinoculation (hpi) with PstAvrRpt2. Substantially higher (approximately 3-fold) amounts of cleaved substrate accumulated in reactions with extracts from pathogen-inoculated than control leaves (Fig. 6*B*).

A localized infection with PstAvrRpt2 induces a generalized immune response throughout the plant—the SAR (2). We tested TOP activity in systemic leaves from plants challenged with a localized inoculation of PstAvrRpt2 and buffer-only infiltrations as control (Fig. 6*A*). We measured a 1.5-fold increase in TOP activity in systemic leaves from PstAvrRpt2-challenged plants compared with controls at 48 hpi (Fig. 6*C*). To ascertain that SAR was triggered in Col-0 by the localized PstAvrRpt2 infiltration, systemic leaves were inoculated with the virulent pathogen PstDC3000 at 48 h postchallenge with PstAvrRpt2 or buffer, followed by measurements of PstDC3000 growth (Fig. 6*D*). As expected, a significant decrease in PstDC3000 growth occurred in plants challenged with PstAvrRpt2 compared with buffer-infiltrated plants at 48 hpi, indicating SAR activation. Control measurements of PstDC3000 titers immediately after leaf inoculation showed no significant differences among plants (Fig. S6*A*). Thus, the TOP activity upsurge correlated with the activation of the local and systemic immunity.

Thiol homeostasis is essential for the activation of plant immunity (46). Although the cellular redox pathways involved in this process are not entirely defined, mutants with defects in the redox homeostasis exhibit immune-deficient phenotypes (47, 48, 49, 50). Plants unable to express the chloroplast-localized NADPH-dependent thioredoxin reductase C (*NTRC*) overaccumulated oxidants and had high susceptibility to pathogenic *P. syringae* strains (51). We hypothesized that *NTRC* might control TOP activity. To test this, we measured TOP activity in two mutants with altered *NTRC* expression—a knockout line (*ntrc*) and an overexpression line (*NTRC*-*OE*) (47, 48). In *ntrc* plants, we found equal TOP activity levels in PstAvrRpt2-challenged and buffer controls; notably, *ntrc* TOP activity reached values similar to those measured in pathogen-challenged Col-0 (Fig. 6*C*), an indication that in the absence of stress, *NTRC* acts as a negative regulator of TOP activity. No significant changes were found in the TOP activity in *OE-NTRC* extracts compared with Col-0 in control or pathogen-challenged plants (Fig. 6*C*).

We tested SAR activation in *ntrc* and *OE-NTRC* lines, as shown in Figure 6*D*. Measurements of PstDC3000 titer immediately after inoculation showed equal inoculum values among genotypes (Fig. S6*A*). However, at 48 hpi, PstDC3000 grew at similar levels in PstAvrRpt2-and buffer-challenged *ntrc* plants indicating that *NTRC* expression is required for SAR execution. On the other hand, overexpression of *NTRC* did not affect SAR, as shown by the wild-type level decrease in PstDC3000 multiplication in *OE-NTRC* plants (Fig. 6*E*). Considering the crucial role of SA-mediated signaling for the oxidative burst and SAR (52, 53), we tested the induction of canonical markers of SA-mediated signaling (*PR1* and *PAD4*), SA synthesis (*ICS1*), and SAR (*SAG13*) in systemic leaves of *ntrc*. Compared with Col-0, the induction of these markers in *ntrc* was significantly suppressed (*PR1* and *SAG13*) or abolished (*ICS1* and *PAD4*) (Fig. 6*F*).

Augmented TOP activity during the plant immune response may result from *TOP1* and *TOP2* induction. Quantification of *TOP1* and *TOP2* mRNA levels in PstAvrRpt2-infiltrated and control leaves showed equal abundance in all samples for both transcripts (Fig. S6*B*); neither *ntrc* nor *OE-NTRC* lines showed changes in *TOPs* mRNA accumulation (Fig. S6*C*). An analysis of *TOP1* and *TOP2* expression patterns in published datasets (https://genevestigator.com/), in Col-0 and mutant lines with altered expression of immune components or defective redox homeostasis, during SAR or oxidative stress (H_2_O_2_), revealed no significant variation in *TOPs* expression levels (Fig. S6*D*).

We reasoned that *TOP1* and *TOP2* participate in the redox signaling activated following the *PstAvrRpt2* localized challenge. We selected for this analysis the plant-specific transcription factors *ZAT10* and *WRKY33*. Both are redox-regulated genes rapidly induced genes in response to ROS accumulation in locally stressed and unstressed systemic tissues of *Arabidopsis* (54); Recently, increased *ZAT10* expression was linked to upregulation of the SA signaling marker *PR1* and the autoimmune response-inducing necrosis, a type of HR-like cell death in wheat (55). *WRKY33* is part of signaling pathways activated by PstDC3000 (56) and necessary for PstAvrRpt2-triggered SAR development (57). We measured *ZAT10* and *WRKY33* induction in Col-0 and *top1top2* mutant, using *PstAvrRpt2*-infiltrated and uninoculated systemic leaves at 48 hpi and as controls, tissue from plants infiltrated with buffer. We found that both *ZAT10* and *WRKY33* were induced at high levels in both local and systemic tissues of Col-0, indicating a role in the *P. syringae*-activated redox signaling (Fig. 6*G*). Notably, in *PstAvrRpt2*-challenged *top1top2* leaves, *ZAT10* and *WRKY33* mRNAs accumulated in significantly higher amounts (∼5-fold and 2.5-fold, respectively) compared with Col-0, whereas the systemic *WRKY33*, but not *ZAT10*, mRNA showed increased accumulation (∼4-fold) relative to wild type (Fig. 6*G*).

### TOP1 and TOP2 cleave a peptide from the immune component ROC1

**Figure 7:**
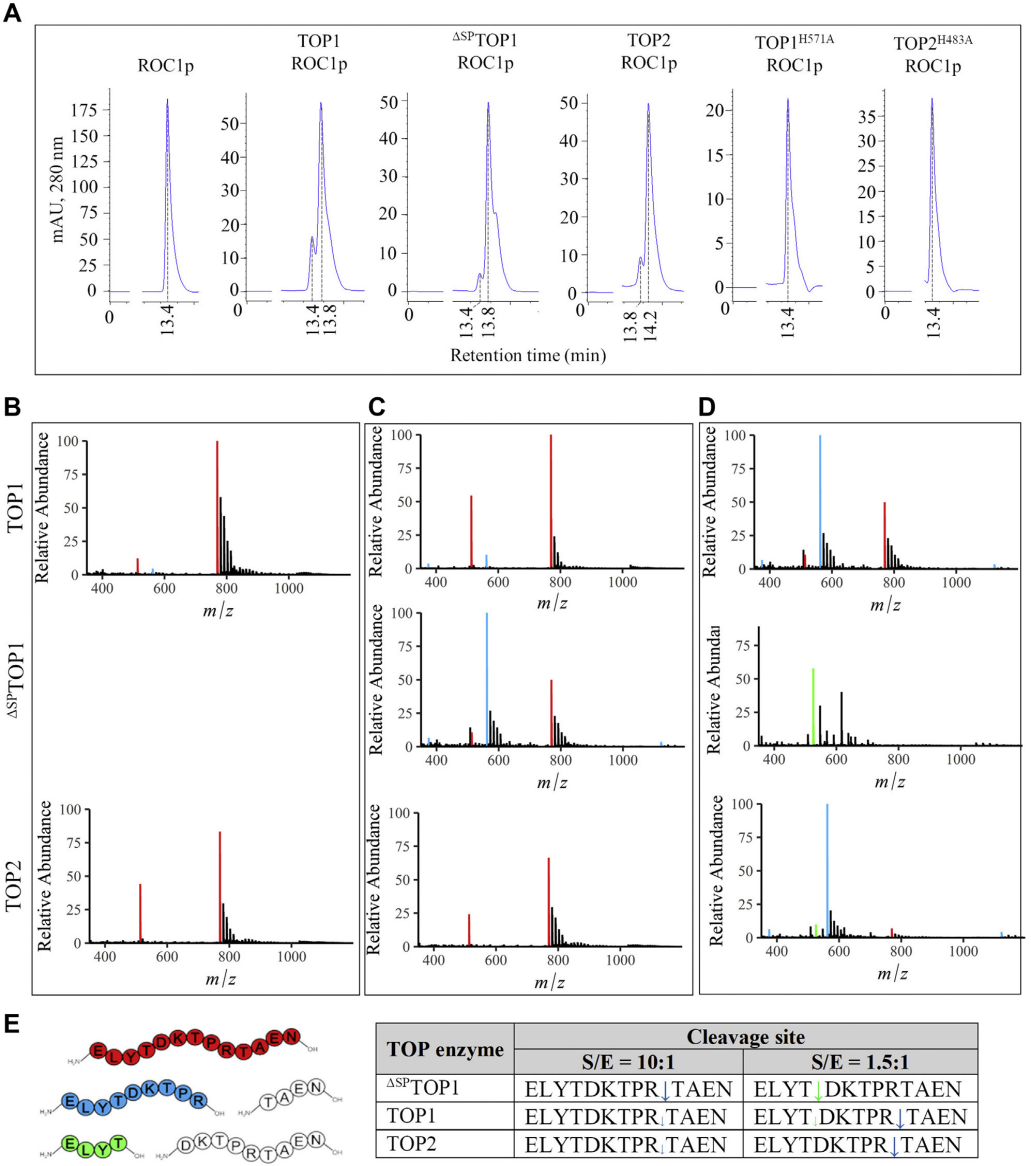
**Cleavage activity of plant TOPs on ROC1.***A*, HPLC chromatograms showing ROC1 peptide (ELYTDKTPRTAEN) elution peaks and retention times in the absence (control) or after incubation with TOP1, ^ΔSP^TOP1, or TOP2. *B*, when ROC1p is treated with the inactive TOP mutants, only the full-length peptide (ELYTDKTPRTAEN, *red*, m/z = 769.38, +2 charge state) was detected in ESI-MS, indicating no cleavage. *C*, at a peptide:TOP reaction mixture ratio of 10:1 one cleavage site was detected for TOP1, ^ΔS^PTOP1, and TOP2. The N-terminal product (ELYTDKTPR, *blue*, m/z = 561.79, +2 charge state) indicates this TOP cleavage site. *D*, at a peptide:TOP reaction mixture ratio of 1.5:1 two cleavage sites were detected for TOP1. The N-terminal product ELYTDKTPR indicates one TOP cleavage site. The N-terminal product (ELYT, *green*, m/z = 525.26, +1 charge state) indicates the second cleavage site. For ^ΔSP^TOP1, the cleavage was indicated by the N-terminal product ELYT, while for TOP2, the cleavage was indicated by the N-terminal product ELYTDKTPR. All observed masses match with the theoretical peptide masses within 2 ppm mass error. The purity of synthesized peptide was determined to be ∼60% through peak area analysis *via* LC-MS. *E*, diagram and table of all TOPs cleavage sites identified within ROC1p.

Plant resistance to PstAvrRpt2 correlated with an upsurge in TOP activity (Fig. 6*A*). TOPs may be acting in the degradation pathway of a critical immune component of the PstAvrRpt2-triggered pathways. To identify possible plant-specific substrates of plant TOPs, we performed a BLAST search of the *Arabidopsis* proteome with a set of known substrates of THOP1 (EC:3.4.24.15) described in (58). We identified one protein, the rotamase cyclophilin 1 (ROC1/CYP18–3), in which the THOP1 (EC:3.4.24.15) cleavage (TA↓EN) site was conserved. Notably, ROC1 is necessary for the self-cleavage of AvrRpt2 protease secreted into plant cells by *P. syringae* and subsequent processing of the immune regulator RIN4 (59). The activity of TOPs on ROC1 was tested using SEC and a ROC1 peptide (ROC1p) containing the THOP1 (EC:3.4.24.15) cleavage site (ELYTDKTPRTA↓EN) (Fig. 7*A* and Table S1). In the control reaction without a TOP enzyme, ROC1p eluted as a single peak (Rt = 13.4 min, according to its molecular weight of 1430 Da). In the presence of TOP1 or ^ΔSP^TOP1, an abundant ROC1p product eluted at Rt = 13.8 min, whereas in the presence of TOP2, a cleaved product eluted at Rt = 14.2 min. To verify that the SEC peaks result from the enzymatic activity of TOPs on ROC1p, we generated two active site mutants. The first His residue in the active site motif HEXXH was replaced with Ala in both TOP1 and TOP2 to generate TOP1^H571A^ and TOP2^H483A^ (Fig. S7); a similar mutation in THOP1 (EC:3.4.24.15) and neurolysin abolished their enzymatic activity on natural substrates. In the presence of TOP1^H571A^ or TOP2^H483A^, ROC1p eluted uncleaved (Fig. 7*A*).

Although all TOPs cleaved ROC1p, their cleavage site selection appeared to be dissimilar. We analyzed reaction mixtures containing TOPs at two different concentrations using electrospray ionization–mass spectrometry (ESI-MS) to determine cleavage site selectivity. In the presence of the inactive TOP1^H571A^ or TOP2^H483A^, ROC1p eluted uncleaved (Fig. 7*B*). At 10:1 of peptide:TOP ratio, the same cleavage site (ELYTDKTPR↓TAEN) was identified for TOP1, ^ΔSP^TOP1, and TOP2; for this cleavage site, the N-terminal product ELYTDKTPR was detected in all reaction mixtures and was most abundant in the ^ΔSP^TOP1 reaction (Fig. 7, *C* and *E*). At the 1.5:1 peptide:TOP ratio, we identified a unique suite of cleavage products for each TOP isoform. TOP1 produced two cleavage sites in ROC1p, including the site identified at 10:1 ratio (ELYTDKTPR↓TAEN) detected by the presence of the abundant product ELYTDKTPR, and a second cleavage site (ELYT↓DKTPRTAEN) detected by the presence of a comparatively smaller amount of the product ELYT (Fig. 7, *D* and *E*). At 1.5:1 with ^ΔSP^TOP1, the ELYT product was detected, resulting from the ELYT↓DKTPRTAEN cleavage; however, TOP2 cleaved ROC1p at the same site identified in the 10:1 reaction (ELYTDKTPR↓TAEN), but more efficiently (Fig. 7, *D* and *E*). Thus, at high substrate concentrations, TOP isoforms cleave ROC1p at PR↓TA with ^ΔSP^TOP1 showing the highest efficiency. At low substrate concentration, PR↓TA is a high-efficiency cleavage site of TOP1 and TOP2, whereas ^ΔSP^TOP1 prefers YT↓DK, which is also cleaved with lower efficiency by TOP1 (Fig. 7*E*).

## Discussion

The biochemical characterization of the *Arabidopsis* TOPs revealed expected similarities and unexpected differences. We confirmed that although both TOPs are zinc-binding enzymes, similar to their mammalian counterparts, TOP2 binds multiple divalent cations with equal or higher affinity. This aspect merits further characterization as the potential functional role of metal selectivity in regulating catalytic activities of metalloproteases has been previously documented (60). An evaluation of TOPs redox-mediated self-interaction in *E. coli* found that both TOP1 and TOP2 monomers were stable under a broad range of redox conditions, and TOP1 was prone to forming higher-MW structures. Nevertheless, we interpreted the *ex vivo* assays' results with prudence, given the nonlinear and concentration-dependent effects of redox treatments on growing *E. coli* in (61), where high oxidative stress caused increased GSSG accumulation. In contrast, lower oxidative stress had nonlinear and even opposite effects on the cells' redox status.

Analyses of the purified TOPs complemented the *ex vivo* analysis by evaluating TOPs intrinsic sensitivity to redox agents. C^52^ and C^611^ were identified as critical residues by site-specific mutagenesis applied to investigate the structural basis of TOP1 dimerization. A mass spectrometric analysis to uncover the thiol status of TOP1 brought evidence that TOP1 forms disulfide-bonded homodimers; all cysteines in the signal peptide participated in disulfide bonds and C^52^ emerged as a participant in all detected disulfides. Measurements of Cys–Cys distances in the crystal structures of ^ΔSP^TOP1 and TOP2 and our TOP1 model do not support the existence of intramolecular disulfides (the primary candidates being the conserved pair of “core” cysteines C^548/460^, C^611/523^ in TOP1/TOP2). However, our observations regarding the stability of Cys-to-Ala TOP1 mutants in *E. coli* suggest the possibility that certain redox conditions facilitate the formation of intramolecular disulfides in TOPs, as observed for other redox-sensitive plant proteins (25). Nevertheless, we cannot rule out the possibility that TOP2 dimerizes as well, as we observed DTT_red_-sensitive dimers of a TOP2-Trx fusion *in vitro* and yeast cells (34). It remains to be seen if, in the plant cell, TOP2 is modified posttranslationally through interactions with more reactive redox sensors such as thioredoxins, or similar to THOP1 (EC:3.4.24.15) (62), undergoes other redox-mediated modifications such as S-glutathionylation. The formation of dimers and multimers in THOP1 (EC:3.4.24.15) was also attributed to intermolecular disulfides (62).

In plants, examples of proteins undergoing thiol–disulfide exchanges are limited; from the known examples, disulfides protect proteins against oxidative inactivation (63), inhibit (64), or have no observable effect (65). Our previous investigations of redox sensitivity of dimeric TOPs showed that the dimer was marginally less active than the monomer and that DTT inhibited their enzymatic activity to a similar extent (34). Thus, similar to TOPs in other eukaryotic systems, dimeric TOP1 maintains its activity; however, a detailed characterization of TOP1's homotypic and heterotypic dimers may reveal differences. For TOP1, dimer formation may represent a regulatory mechanism in high oxidation environments through SA binding (41) and SA-mediated TOP1 dimerization (34); dimerization *via* transit peptide cysteines may also interfere with TOP1 transport into organelles by blocking its phosphorylation (66) or blocking interactions with translocons (67). Besides chloroplasts, TOP1 was detected in the proteomes of the cytosol (68), apoplast (69), and the plasma membrane following ETI activation by PstAvrRpt2 (70); thus, TOP1 may contribute to proteolysis in multiple subcellular compartments.

Measurements of subcellular thiol redox dynamics indicate that the plant cell cytosol is highly reducing (–320 mV); the estimated redox poise in mitochondria and light-exposed chloroplasts is even more negative (–380 mV) (71). Conversely, in the dark, when the strong reductive power of photosynthesis is missing, chloroplasts' redox environment adjusts to lower potentials (*i.e.,* up to –240 mV) (72, 73). The redox poise of the cytosol and organelles undergoes dramatic shifts during an immune response (74), and the oxidative burst is a feature of both the local and systemic immunity (6, 75). We determined that oxidation increased TOPs activity; the estimated TOP1 midpoint redox potential of –200 mV renders it active in more oxidative environments, suggesting a role during the pathogen-triggered oxidation waves. Its E_m_ value is within the range of other redox-active proteins such as thioredoxins and thioredoxin-regulated enzymes (42). Multiple cysteines in the transit peptide and peptidase domain of TOP1 contributed to its oxidative activation, suggesting that cytosolic and organellar isoforms are both subject to redox regulation. On the other hand, the unique C^405^ was the most robust determinant of TOP2 oxidative activation. Enzyme activation by thiol oxidation plays a mechanistic role in many proteins. Activation of enzymes in the autophagy pathway during oxidative stress efficiently removed oxidized proteins (76); oxidation activated the cyclooxygenase COX (77) and the plant OXI1 necessary for redox-mediated signaling and pathogen resistance (78).

Consistent with the positive effect of oxidation on TOPs *in vitro* activity, we found that activation of local and systemic immunity correlated with enhanced TOP activity in the plant. Notably, *TOP1* and *TOP2* expression did not change significantly under various treatments and in mutant genotypes. Although we cannot account for possible variability in TOPs levels due to posttranscriptional events, the correlation between transcript and protein accumulation is high in *Arabidopsis* leaves (∼70%, according to (79)). The study of *NTRC* mutants further supports the hypothesis that TOP activity is under redox regulation in the plant. The oxidative burst increases cellular oxidative potential, leading to TOP activity upsurge; the increased TOP activity of the nonstressed *ntrc* plants may be a consequence of the mutant's increased ROS accumulation (51) and deficient thiol homeostasis (80). We postulate that NTRC, or a downstream component in the NTRC-mediated signaling, maintains TOPs in a reduced, low-activity state. Although our knowledge of the rich repertoire of plant thioredoxins is expanding, little is known about the range of oxidized residues they target, substrate selectivity, redox potentials, and specificity in redox-mediated pathways (81, 82). Notably, we found that *ntrc* plants cannot activate SA signaling and SAR, although it is unclear if these defects are a cause or consequence of defective redox regulation of TOPs. In the *top1top2* knockout line, induction of ROS marker genes was deregulated, with an overall increase in local and systemic gene activity. These results point to an adverse effect of *TOPs* on ROS-responsive gene induction. The elevated redox marker expression in *top1top2* is consistent with its augmented hypersensitive response and cell death (25, 28). These results support a role for *TOP1* and *TOP2* to SAR regulation through the modulation of redox-sensitive pathways. To begin disentangling TOPs role in immune redox signaling, to this end, we searched for immune-associated TOP substrates. TOPs cleaved the key immune component, ROC1, at sites different from the THOP1's, and site selection was dependent on the substrate's concentration. ROC1 plays an essential role in plant–pathogen recognition by promoting the *P. syringa*e AvrRpt2 protease maturation; this effector protein is secreted in plant cells during infection and activates ETI following its recognition by the RPS2 immune receptor (59). Although classified as a cytosolic rotamase (83), ROC1 was identified in the plastid stroma (84), mitochondria (85), plasma membrane (86, 87), and extracellular proteome (69). The importance of TOP1 and TOP2 proteolysis of ROC1 for the plant immune response remains to be explored.

We rationalized *in vitro* and *in vivo* observations to signify that changes in the plant cell's redox environment modify sensitive TOPs thiols and affect their activity. Reducing conditions prevalent in nonstressed plants during the light cycle would keep TOPs activity at low levels; oxidative stress triggered by pathogen infection and in nonphotosynthesizing chloroplasts would augment TOPs activity *via* thiol oxidation. Increased TOP activity in systemic tissues may contribute to immune priming, whereby specific cellular defense responses are operationally prepared for augmented expression upon pathogen attack but with yet little-understood molecular mechanisms (88). Further studies of TOP substrates and downstream signaling pathways may reveal a more widespread role of redox-mediated proteolysis on plant immunity.

## Experimental procedures

### Molecular modeling and biochemical analyses

Chimera (89, 90) was used to visualize and analyze the known molecular structures of ^ΔSP^TOP1 and TOP2 and to obtain a model of the full-length TOP1 and calculate the Cys–Cys distances within the 3D structures. For the thermal denaturation of TOPs, time-resolved intensity decay for tryptophan was measured using a spectrofluorometric instrument Olis DM 45 at 280 nm excitation and 300–500 nm for the fluorescence emission.

### Cloning and site-directed mutagenesis

The cDNA sequences encoding *A. thaliana TOP1* (AT5G65620) and *TOP2* (AT5G10540) were cloned into pET-28a (Agilent) in a translational frame with 6xHis N-terminal tags. Cysteine mutagenesis was performed by site-directed mutagenesis, using the wild-type cDNAs, gene-specific primers, and Phusion High-Fidelity DNA Polymerase (New England Biolabs). The PCR products were separated on agarose gels (0.8%), purified using the QIAquick gel extraction Kit (Qiagen), and cloned into pET-28a using NheI and SalI restriction enzymes (NEB) and T4 DNA ligase (NEB). The plasmids were used for the expression of TOPs in *E. coli*. Additional details are described in Supplemental Methods.

### Protein expression and purification

Plasmids containing the native and mutant TOPs were transformed into competent *E. coli* NiCo21 (DE3) cells (NEB). *E. coli* cultures were grown with shaking at 37 °C in LB Broth media in the presence of 50 mg/L kanamycin. At OD_600_ around 0.5–0.6, the cultures were induced with Isopropyl β-D-1-thiogalactopyranoside (IPTG) at 100 mg/L and collected after 4 h. Pellets were stored at –80 °C for at least 1 day, then thawed on ice, and lysed in B-PER complete bacterial protein extraction reagent (ThermoFisher) before processing. The lysed cells were centrifuged at 4000 rpm for 30 min at 4 °C. The supernatants were concentrated in an Amicon stirred cell (50 ml) with Ultrafiltration discs (PLGC, Ultracel regenerated cellulose, 30 kDa) and then purified *via* batch HisPur Ni-NTA Resin (Thermo Fisher Scientific) following the manufacturer's protocol. Before analysis, proteins were dialyzed overnight with 50 mM Tris buffer containing 100 mM NaCl at pH 8.0. Purified proteins were aliquoted and stored in 20% glycerol at –80 °C.

### Ion binding assays

The concentration of ions (Zn(II), Cu(II), Ni(II), Co(II), Mn(II)) in purified TOPs proteins was determined using an inductively coupled plasma–mass spectrophotometer (ICP-MS) (PerkinElmer SCIEX, ELAN DRC II), which has a limit of quantification of 1 μg/L, as described in (91, 92).

### PAGE electrophoresis and immunoblotting

Bacterial cultures expressing wild-type and mutant TOPs were grown in Luria–Bertani (LB) medium containing kanamycin (50 mg/L) to OD_600_ = 0.6 induced 4 h with 100 mg/L IPTG. *E. coli* cells were collected by centrifugation, and pellets were lysed using B-PER complete bacterial protein extraction reagent. The Bradford assay determined protein concentration before equal amounts of proteins were loaded into PAGE gels and run using a BIO-RAD electrophoresis system. Proteins were transferred to polyvinylidene difluoride (PVDF) membranes using the semidry transfer method at 20 V for 40 min, blocked in 5% nonfat milk, probed with the primary antibody (6x-His Tag Monoclonal Antibody) (1:500), and then the secondary antibody Goat anti-Mouse IgG (H + L) HRP conjugated (1:5000) (Thermo Fisher) at room temperature. Membranes were incubated with ECL substrate (Bio-Rad), and the bands were visualized using a Bio-Rad imaging system. For native PAGE, purified TOPs were mixed with Native PAGE 4× sample loading buffer to a final concentration 1×, loaded in a native PAGE, and ran at 200 V using a Bio-Rad system. PageBlue stain (Thermo Scientific) was used to visualize protein bands as described by the manufacturer. For immunoblotting, proteins were transferred to PVDF membranes and processed as described.

### Redox treatments

For *in vivo* redox treatments, *E. coli* cultures were grown with shaking at 37 °C in LB in the presence of 50 mg/L kanamycin, induced for 4 h with IPTG (100 mg/L), and collected at OD_600_ of 0.5–0.6. In total, 250 μl of 20 mM reducing and oxidizing agents was added to 2.750 ml of *E. coli* culture and grown for a further 10 h. The samples were collected and stored at –20 °C before being used for native or reducing PAGE and immunoblotting. For *in vitro* redox treatments, purified proteins were dialyzed against 50 mM Tris-buffer pH 7.0 using membrane tubes (6–8 kDa) at 4 °C overnight and concentrated before 30 min-long treatments with a 10-fold molar excess of oxidative and reductive agents as described in (39). The reducing agents used were L-Glutathione reduced (GSH) and 1, 4-Dithiothreitol (DTT_red_); the oxidizing agents were L-Glutathione oxidized (GSSG), trans-4, 5-dihydroxy-1, 2-dithiane (DTT_ox_), and hydrogen peroxide (H_2_O_2_). To obtain a range of redox potentials, GSH, GSSG, DTT_red,_ and DTT_ox_ were mixed in defined ratios (39). Additional details are described in Supplemental Methods. We analyzed the dependence of TOP1 activity on the redox potential using DTT_red_/DTT_ox_ and GSH/GSSG. E_m_ values were estimated by fitting oxidation ratio (R) calculated with R = F_ox_/(F_ox_ + F_red_) (F_ox_ and F_red_ are the fraction of enzyme at oxidized and reduced conditions, respectively) to calculated potentials of redox couples using Nernst equation, E_h_ = E_m_ + (RT/nF)(ln([F_ox_]/[F_red_])), with RT/F = 25.693 mV and n=number of electrons exchanged in the redox reaction. The enzyme activity reduction ratio was estimated from fluorescence measurements of enzyme activity over the substrate (initial reaction rates). The fitting equation (Y = 1/(1 + exp-(X − E_m_)∗n/25.693)) was implemented using GraphPad PRISM v8 as a nonlinear least square fitting method. Calculation of TOPs E_m_ value is based on the equilibrium midpoint potential values of DTTred/DTTox redox couple (−327 mV) and GSH/GSSG redox couple (−240 mV) at pH 7.0.

### Enzymatic activity assays

TOP enzymatic activity was measured by using an Olis DM 45 spectrofluorometric instrument. Purified TOPs were concentrated to 10 μg and incubated in 500 μl of 50 mM Tris-buffer pH 7.0, supplemented with 100 mM NaCl, with 8 μM of the fluorogenic substrate peptide Mca-PLGPK(DNP)-OH (Mca–7-methoxycoumarin-4-acetyl–DNP–2,4-dinitrophenol) (Enzo Lifesciences) or the ROC1 peptide (AB Clonal). The cleavage of the fluorogenic substrate was monitored by λ_ex_ at 330 nm and λ_em_ at 400 nm, and TOP activity measurements in plant extracts were done as described in (28). We measured the initial reaction rate (μM s^−1^) of TOPs activity from the *in vitro* reactions (first 100 s time points) using methods implemented in a recently developed online tool (93). We used the logarithmic approximation method (94), except for controls, where we used a linear method (95), to minimize the standard error for the time interval of measurements. TOP1 and TOP2 progression curves of reaction product (cleaved substrate concentration) were measured at a range of redox potentials. In tissue lysates, TOP activity was estimated by calculating the normalized total reaction product (integrated using the area under curve tool in GraphPad PRISM v8 [https://www.graphpad.com/scientific-software/prism/]) per time unit. The product reaction concentration was normalized per microgram of total protein.

### Plant material and growth conditions

*A. thaliana* ecotype Columbia (Col-0), *NTRC*-*OE*, and *ntrc* mutant seeds were sterilized as described in (96), grown on MS media for 10 days, then transferred to a controlled growth chamber with a 12 h light and 12 h dark setup, 100 μmol m^−2^ s^−1^ photon flux density, and relative humidity of 60 to 65%. Day and night temperatures were set to 23 °C and 21 °C, respectively.

### Pathogen assays

*P. syringae* pv. *tomato* (*Pst*) strain DC3000c AvrRpt2 was cultivated at 28 °C in King's B medium. Pathogen assays were performed as described in (97). Briefly, overnight log-phase cultures were diluted to final optical densities (OD_600_) for leaf inoculations. For ETI, PstAvrRpt2 was suspended in MgCl_2_ (1 × 10^5^ CFU/ml) before infiltration in the leaves of mature plants. For SAR, one lower (1°) leaf was infiltrated with a *Pst*AvrRpt2 suspension of 1 × 10^7^ CFU/ml; 48 h postprimary infiltration, upper leaves were harvested for the determination of *TOPs* expression and activity or infiltrated with PstDC3000 (1 × 10^5^ CFU/ml). Experiments were performed with 4-week-old plants with a uniform appearance.

### Quantitative PCR analysis

Total RNA was extracted using the RNA extraction kit (Sigma Aldrich). One microgram of total RNA was used for cDNA synthesis with the iScript gDNA Clear cDNA Synthesis kit (Bio-Rad). Transcript levels were measured using SYBR Green technology and EvaGreen qPCR mix plus (Solis BioDyne). Data were analyzed using the ^ΔΔ^CT method (98) and the endogenous control ubiquitin.

### High-performance liquid chromatography (HPLC)

His-tagged TOP1 and TOP2 purified from *E. coli* and dialyzed were treated with redox agents with gentle mixing for 30 min at RT. The redox reagents have a strong absorbance at 280 nm; therefore, after treatments and before HPLC, proteins were subjected to second purification and redialyzed in 50 mM Tris buffer at pH 7.0 supplemented with 100 mM NaCl to avoid protein aggregation. The HPLC was performed using an XBridge BEH200A SEC 3.5 μm 7.8 × 300 mm column connected to an Agilent 1100 series HPLC system. The column was equilibrated with HPLC water for 30 min, followed by 50 mM Sodium phosphate buffer containing 150 mM NaCl and 0.02% NaN_3_ at pH 7.00, for 30 min. The isocratic mode was used to run the sample for 30 min with 50 mM sodium phosphate buffer with 150 mM NaCl and 0.02% NaN_3_, at a flowrate of 0.88 μl/min. Chromatograms were recorded and processed with the Agilent HPLC software B.04.03.

### In-gel digestion and LC-MS/MS analysis

The native PAGE gel was excised manually around the masses for the TOP1 monomer and dimer for downstream processing. Gel slices were destained three times with 300 μl of 50 mM ammonium bicarbonate/50% ACN solution before in-gel trypsin digestion (5 μl of 0.5 μg/μl trypsin in 50 mM acetic acid) was performed in 50 mM ammonium bicarbonate overnight at RT. Following trypsin digestion, 10 mM calcium chloride was added, and in-gel chymotrypsin digestion (5 μl of 0.5 μg/μl chymotrypsin in 1 mM hydrochloric acid) was performed in the same solution for 4 h at RT. Peptides were extracted first with 30 μl of 1% formic acid/2% ACN, followed by 30 μl of 60% ACN. Peptides were dried by vacuum centrifugation, resuspended in 1 ml of 0.1% TFA, and desalted by reversed-phase solid-phase extraction as described above before LC-MS/MS. Samples were resuspended in 5% acetonitrile/0.1% TFA and analyzed using an Acquity UPLC M-Class System (Waters) coupled to a Q Exactive HF-X mass spectrometer (Thermo Fisher Scientific). Mobile phase A consisted of water with 0.1% formic acid (Thermo Fisher Scientific), and mobile phase B was acetonitrile with 0.1% formic acid. Injections were made to a Symmetry C18 trap column (100 Å, 5 μm, 180 μm × 20 mm; Waters) with a flow rate of 5 μl/min for 3 min using 99% A and 1% B. Peptides were then separated on an HSS T3 C18 column (100 Å, 1.8 μm, 75 μm × 250 mm; Waters) using a linear gradient of increasing mobile phase B at a flow rate of 300 nl/min. Mobile phase B was held at 5% for 1 min, then increased from 5% to 50% in 30 min before ramping to 85% in 2 min, where it was held for 3 min before returning to 5% in 1 min and re-equilibrating for 23 min. The mass spectrometer was operated in positive polarity, and the Nanospray Flex source had spray voltage floating at 2.1 kV, the capillary temperature at 320 °C, and funnel RF level at 40. MS survey scans were collected with a scan range of 350–2000 m/z at a resolving power of 120,000 and an AGC target of 3 × 106 with a maximum injection time of 50 ms. A top 20 data-dependent acquisition was used where HCD fragmentation of precursor ions having +2 to +7 charge state was performed using a normalized collision energy setting of 28. MS/MS scans were performed at a resolving power of 30,000 and an AGC target of 1 × 105 with a maximum injection time of 100 ms. Dynamic exclusion for precursor m/z was set to a 10 s window.

### Proteomics database searching

Acquired spectral files (∗.raw) were converted into combined peak lists (∗.mgf) using MSConvertGUI (version 3.0.18130-e9d0c75b5) (99) for peptide sequence determination by Mascot (Matrix Science, version 2.5.1). Database searching was performed against the *A. thaliana* UniProt database (https://www.uniprot.org/proteomes/UP000006548, 39,359 canonical entries, accessed April 24, 2020) and *E. coli* UniProt database (https://www.uniprot.org/proteomes/UP000002032, 4156 canonical entries, accessed December 4, 2020) with sequences for common laboratory contaminants (https://www.thegpm.org/cRAP/, 116 entries, accessed April 24, 2020) appended. Target-decoy searches of MS/MS data used a trypsin (specificity: K/R that are not before P)/chymotrypsin (specificity: F/Y/W/L that are not before P) protease specificity with the possibility of two missed cleavages, peptide/fragment mass tolerances of 15 ppm/0.02 Da, and variable modifications of N-terminus acetylation and methionine oxidation. Significant peptide identifications above the identity or homology threshold were adjusted to less than 1% peptide FDR using the embedded Percolator algorithm (100). The threshold for a peptide–spectrum match was Percolator-adjusted Mascot Peptide Score >13.

### *In vitro* enzyme assay, solid-phase extraction, and ESI-MS analysis

The custom synthesized ROC1 peptide ELYTDKPRTAEN (AB Clonal) sequence was solubilized in 500 μl 50 mM Tris, pH 7.5. To initiate the enzyme assay, TOP1, ^ΔSP^TOP1, TOP2, TOP^H571D^, or TOP2^H483D^ was added at a peptide:TOP ratio of 1.5:1 and 10:1. The reaction mixture was incubated at 23 °C for 30 min. Before ESI-MS analysis, desalting was performed using 50 mg/1.0 ml Sep-Pak C18 cartridges (Waters) held in an SPE 24-position vacuum manifold (Phenomenex) at a flow rate of 1 drop/s. Resin was first pre-eluted using 1 ml of 80% acetonitrile/0.1% TFA before equilibration with 1 ml of 0.1% TFA. Samples were acidified to pH 3 using 10% TFA and loaded onto the cartridges in two passes, and then washed using 1 ml of 0.1% TFA. Peptides were eluted using 1 ml of 80% acetonitrile/0.1% TFA and concentrated by vacuum centrifugation to dryness. Samples were resuspended in 100 μl of 50% methanol/0.1% formic acid. Peptides were directly infused *via* ESI on a Thermo Q Exactive HF-X Hybrid mass spectrometer for intact mass analysis. Samples were injected at a flow rate of 10 μl/min, and full MS scans were analyzed in the Orbitrap. The mass spectrometer was operated at a resolving power of 120,000, positive polarity, spray voltage of 3 kV, with 150–2000 *m/z* range, and collecting 100 scans per sample for averaging.

## Data availability

The mass spectrometry proteomics data have been deposited to the ProteomeXchange Consortium *via* the PRIDE partner repository and can be accessed with the dataset identifier PXD024059 and 10.6019/PXD024059. Username: reviewer_pxd024059@ebi.ac.uk; password: zhjMfoSh.

## Supporting information

This article contains supporting information (39).

## Conflict of interest

The authors declare that they have no conflicts of interest with the contents of this article.

## References

[1] Axtell M.J., Staskawicz B.J. (2003). Initiation of RPS2-specified disease resistance in Arabidopsis is coupled to the AvrRpt2-directed elimination of RIN4. Cell.

[2] Grant M., Lamb C. (2006). Systemic immunity. Curr. Opin. Plant Biol..

[3] Durrant W.E., Dong X. (2004). Systemic acquired resistance. Annu. Rev. Phytopathol..

[4] Czarnocka W., Karpiński S. (2018). Friend or foe? Reactive oxygen species production, scavenging and signaling in plant response to environmental stresses. Free Radic. Biol. Med..

[5] Morales J., Kadota Y., Zipfel C., Molina A., Torres M.-A. (2016). The Arabidopsis NADPH oxidases RbohD and RbohF display differential expression patterns and contributions during plant immunity. J. Exp. Bot..

[6] Alvarez M.E., Pennell R.I., Meijer P.-J., Ishikawa A., Dixon R.A., Lamb C. (1998). Reactive oxygen intermediates mediate a systemic signal network in the establishment of plant immunity. Cell.

[7] El-Shetehy M., Wang C., Shine M.B., Yu K., Kachroo A., Kachroo P. (2015). Nitric oxide and reactive oxygen species are required for systemic acquired resistance in plants. Plant Signal. Behav..

[8] Lee S.J., Kim D.-G., Lee K.-Y., Koo J.S., Lee B.-J. (2018). Regulatory mechanisms of thiol-based redox sensors: Lessons learned from structural studies on prokaryotic redox sensors. Arch. Pharm. Res..

[9] Reniere M.L. (2018). Reduce, induce, thrive: Bacterial redox sensing during pathogenesis. J. Bacteriol..

[10] Noctor G. (2006). Metabolic signalling in defence and stress: The central roles of soluble redox couples. Plant Cell Environ..

[11] Hammerschmidt R., Nuckles E.M., Kuć J. (1982). Association of enhanced peroxidase activity with induced systemic resistance of cucumber to Colletotrichum lagenarium. Physiol. Plant Pathol..

[12] Chai H.B., Doke N. (1987). Systemic activation of O-2 generating reaction, superoxide dismutase, and peroxidase in potato plants in relation to induction of systemic resistance to phytophthora infestans. Jpn. J. Phytopathol..

[13] Gadjev I., Vanderauwera S., Gechev T.S., Laloi C., Minkov I.N., Shulaev V., Apel K., Inzé D., Mittler R., Van Breusegem F. (2006). Transcriptomic footprints disclose specificity of reactive oxygen species signaling in Arabidopsis. Plant Physiol..

[14] Rosenwasser S., Fluhr R., Joshi J.R., Leviatan N., Sela N., Hetzroni A., Friedman H. (2013). ROSMETER: A bioinformatic tool for the identification of transcriptomic imprints related to reactive oxygen species type and origin provides new insights into stress responses. Plant Physiol..

[15] Pitzschke A., Djamei A., Bitton F., Hirt H. (2009). A major role of the MEKK1–MKK1/2–MPK4 pathway in ROS signalling. Mol. Plant.

[16] Dietz K.-J., Mittler R., Noctor G. (2016). Recent progress in understanding the role of reactive oxygen species in plant cell signaling. Plant Physiol..

[17] Grant J.J., Loake G.J. (2000). Role of reactive oxygen intermediates and cognate redox signaling in disease resistance. Plant Physiol..

[18] Gaupels F., Durner J., Kogel K. (2017). Production, amplification and systemic propagation of redox messengers in plants? The phloem can do it all!. New Phytol..

[19] Waszczak C., Akter S., Eeckhout D., Persiau G., Wahni K., Bodra N., Van Molle I., De Smet B., Vertommen D., Gevaert K. (2014). Sulfenome mining in Arabidopsis thaliana. Proc. Natl. Acad. Sci. U. S. A..

[20] Liebthal M., Maynard D., Dietz K.-J. (2018). Peroxiredoxins and redox signaling in plants. Antioxid. Redox Signal..

[21] Tada Y., Spoel S.H., Pajerowska-Mukhtar K., Mou Z., Song J., Wang C., Zuo J., Dong X. (2008). Plant immunity requires conformational charges of NPR1 via S-nitrosylation and thioredoxins. Science.

[22] Suhm T., Kaimal J.M., Dawitz H., Peselj C., Masser A.E., Hanzén S., Ambrožič M., Smialowska A., Björck M.L., Brzezinski P. (2018). Mitochondrial translation efficiency controls cytoplasmic protein homeostasis. Cell Metab..

[23] Samant R.S., Livingston C.M., Sontag E.M., Frydman J. (2018). Distinct proteostasis circuits cooperate in nuclear and cytoplasmic protein quality control. Nature.

[24] Majsec K., Bhuiyan N.H., Sun Q., Kumari S., Kumar V., Ware D., van Wijk K.J. (2017). The plastid and mitochondrial peptidase network in Arabidopsis thaliana: A foundation for testing genetic interactions and functions in organellar proteostasis. Plant Cell..

[25] McConnell E.W., Berg P., Westlake T.J., Wilson K.M., Popescu G.V., Hicks L.M., Popescu S.C. (2019). Proteome-wide analysis of cysteine reactivity during effector-triggered immunity. Plant Physiol..

[26] Tisljar U. (1993). Thimet oligopeptidase—a review of a thiol dependent metallo-endopeptidase also known as Pz-peptidase endopeptidase 24.15 and endo-oligopeptidase. Biol. Chem. Hoppe-seyler.

[27] Malvezzi A., Higa P.M., Antonia T., do Amaral G.M.S., Gozzo F.C., Ferro E.S., Castro L.M., de Rezende L., Monteiro G., Demasi M. (2012). The cysteine-rich protein thimet oligopeptidase as a model of the structural requirements for S-glutathiolation and oxidative oligomerization. PLoS One.

[28] Moreau M.W., Timothy Z., Giulio P., George T., Miaoying N., Christos P.S. (2013). The Arabidopsis oligopeptidases TOP1 and TOP2 are salicylic acid targets that modulate SA-mediated signaling and the immune response. Plant J..

[29] Kmiec B., Teixeira P.F., Berntsson R.P.-A., Murcha M.W., Branca R.M.M., Radomiljac J.D., Regberg J., Svensson L.M., Bakali A., Langel Ü. (2013). Organellar oligopeptidase (OOP) provides a complementary pathway for targeting peptide degradation in mitochondria and chloroplasts. Proc. Natl. Acad. Sci. U. S. A..

[30] Kmiec B., Teixeira P.F., Glaser E. (2014). Shredding the signal: Targeting peptide degradation in mitochondria and chloroplasts. Trends Plant Sci..

[31] Ito J., Batth T.S., Petzold C.J., Redding-Johanson A.M., Mukhopadhyay A., Verboom R., Meyer E.H., Millar A.H., Heazlewood J.L. (2011). Analysis of the Arabidopsis cytosolic proteome highlights subcellular partitioning of central plant metabolism. J. Proteome Res..

[32] Polge C., Jaquinod M., Holzer F., Bourguignon J., Walling L., Brouquisse R. (2009). Evidence for the existence in Arabidopsis thaliana of the proteasome proteolytic pathway activation in response to cadmium. J. Biol. Chem..

[33] Wang R., Rajagopalan K., Sadre-Bazzaz K., Moreau M., Klessig D.F., Tong L. (2014). Structure of the Arabidopsis thaliana TOP2 oligopeptidase. Acta Crystallogr. Sect. F Struct. Biol. Commun..

[34] Westlake T.J., Ricci W.A., Popescu G.V., Popescu S.C. (2015). Dimerization and thiol sensitivity of the salicylic acid binding thimet oligopeptidases TOP1 and TOP2 define their functions in redox-sensitive cellular pathways. Front. Plant Sci..

[35] Eswar N., Eramian D., Webb B., Shen M.-Y., Sali A. (2008). Protein structure modeling with MODELLER. Structural Proteomics, Part of the Methods in Molecular Biology™ book series (MIMB, volume 426).

[36] Villafranca J.E., Howell E.E., Oatley S.J., Xuong N.H., Kraut J. (1987). An engineered disulfide bond in dihydrofolate reductase. Biochemistry.

[37] Grasso G., Magrì A., Bellia F., Pietropaolo A., La Mendola D., Rizzarelli E. (2014). The copper(II) and zinc(II) coordination mode of HExxH and HxxEH motif in small peptides: The role of carboxylate location and hydrogen bonding network. J. Inorg. Biochem..

[38] Nampoothiri K.M., Nagy V., Kovacs K., Szakacs G., Pandey A. (2005). l-leucine aminopeptidase production by filamentous Aspergillus fungi. Lett. Appl. Microbiol..

[39] Zannini F., Couturier J., Keech O., Rouhier N. (2017). *In vitro* alkylation methods for assessing the protein redox state. Photorespiration.

[40] Chib R., Butler S., Raut S., Shah S., Borejdo J., Gryczynski Z., Gryczynski I. (2015). Effect of quencher, denaturants, temperature and pH on the fluorescent properties of BSA protected gold nanoclusters. J. Lumin..

[41] Moreau M., Westlake T., Zampogna G., Popescu G., Tian M., Noutsos C., Popescu S. (2013). The Arabidopsis oligopeptidases TOP1 and TOP2 are salicylic acid targets that modulate SA-mediated signaling and the immune response. Plant J..

[42] Hirasawa M., Schürmann P., Jacquot J.-P., Manieri W., Jacquot P., Keryer E., Hartman F.C., Knaff D.B. (1999). Oxidation-reduction properties of chloroplast thioredoxins, ferredoxin: Thioredoxin reductase, and thioredoxin f-regulated enzymes. Biochemistry.

[43] Hicks L.M., Cahoon R.E., Bonner E.R., Rivard R.S., Sheffield J., Jez J.M. (2007). Thiol-based regulation of redox-active glutamate-cysteine ligase from Arabidopsis thaliana. Plant Cell..

[44] Moreau M., Westlake T., Zampogna G., Popescu G., Tian M., Noutsos C., Popescu S. (2013). The A rabidopsis oligopeptidases TOP 1 and TOP 2 are salicylic acid targets that modulate SA-mediated signaling and the immune response. Plant J..

[45] Iannetta A., Rogers H.T., Al-Mohanna T., O’Brien J.N., Wommack A.J., Popescu S.C., Hicks L.M. (2021). Profiling thimet oligopeptidase-mediated proteolysis in Arabidopsis thaliana. Plant J..

[46] Álvarez C., Ángeles Bermúdez M., Romero L.C., Gotor C., García I. (2012). Cysteine homeostasis plays an essential role in plant immunity. New Phytol..

[47] Toivola J., Nikkanen L., Dahlström K.M., Salminen T.A., Lepistö A., Vignols F., Rintamäki E. (2013). Overexpression of chloroplast NADPH-dependent thioredoxin reductase in Arabidopsis enhances leaf growth and elucidates *in vivo* function of reductase and thioredoxin domains. Front. Plant Sci..

[48] Lepistö A., Kangasjärvi S., Luomala E.-M., Brader G., Sipari N., Keränen M., Keinänen M., Rintamäki E. (2009). Chloroplast NADPH-thioredoxin reductase interacts with photoperiodic development in Arabidopsis. Plant Physiol..

[49] Michalska J., Zauber H., Buchanan B.B., Cejudo F.J., Geigenberger P. (2009). NTRC links built-in thioredoxin to light and sucrose in regulating starch synthesis in chloroplasts and amyloplasts. Proc. Natl. Acad. Sci. U. S. A..

[50] Pulido P., Spínola M.C., Kirchsteiger K., Guinea M., Pascual M.B., Sahrawy M., Sandalio L.M., Dietz K.-J., González M., Cejudo F.J. (2010). Functional analysis of the pathways for 2-Cys peroxiredoxin reduction in Arabidopsis thaliana chloroplasts. J. Exp. Bot..

[51] Ishiga Y., Ishiga T., Ikeda Y., Matsuura T., Mysore K.S. (2016). NADPH-dependent thioredoxin reductase C plays a role in nonhost disease resistance against Pseudomonas syringae pathogens by regulating chloroplast-generated reactive oxygen species. PeerJ.

[52] Gaffney T., Friedrich L., Vernooij B., Negrotto D., Nye G., Uknes S., Ward E., Kessmann H., Ryals J. (1993). Requirement of salicylic acid for the induction of systemic acquired resistance. Science.

[53] Chen Z., Silva H., Klessig D.F. (1993). Active oxygen species in the induction of plant systemic acquired resistance by salicylic acid. Science.

[54] Miller G., Schlauch K., Tam R., Cortes D., Torres M.A., Shulaev V., Dangl J.L., Mittler R. (2009). The plant NADPH oxidase RBOHD mediates rapid systemic signaling in response to diverse stimuli. Sci. Signal..

[55] Kuki Y., Ohno R., Yoshida K., Takumi S. (2020). Heterologous expression of wheat WRKY transcription factor genes transcriptionally activated in hybrid necrosis strains alters abiotic and biotic stress tolerance in transgenic Arabidopsis. Plant Physiol. Biochem..

[56] Qiu J., Fiil B.K., Petersen K., Nielsen H.B., Botanga C.J., Thorgrimsen S., Palma K., Suarez-Rodriguez M.C., Sandbech-Clausen S., Lichota J. (2008). Arabidopsis MAP kinase 4 regulates gene expression through transcription factor release in the nucleus. EMBO J..

[57] Wang Y., Schuck S., Wu J., Yang P., Döring A.-C., Zeier J., Tsuda K. (2018). A MPK3/6-WRKY33-ALD1-pipecolic acid regulatory loop contributes to systemic acquired resistance. Plant Cell..

[58] Oliveira V., Campos M., Melo R.L., Ferro E.S., Camargo A.C.M., Juliano M.A., Juliano L. (2001). Substrate specificity characterization of recombinant metallo oligo-peptidases thimet oligopeptidase and neurolysin. Biochemistry.

[59] Coaker G., Falick A., Staskawicz B. (2005). Activation of a phytopathogenic bacterial effector protein by a eukaryotic cyclophilin. Science.

[60] Fukasawa K.M., Hata T., Ono Y., Hirose J. (2011). Metal preferences of zinc-binding motif on metalloproteases. J. Amino Acids.

[61] Smirnova G.V., Muzyka N.G., Glukhovchenko M.N., Oktyabrsky O.N. (2000). Effects of menadione and hydrogen peroxide on glutathione status in growing Escherichia coli. Free Radic. Biol. Med..

[62] Demasi M., Piassa Filho G.M., Castro L.M., Ferreira J.C., Rioli V., Ferro E.S. (2008). Oligomerization of the cysteinyl-rich oligopeptidase EP24. 15 is triggered by S-glutathionylation. Free Radic. Biol. Med..

[63] Tossounian M.-A., Van Molle I., Wahni K., Jacques S., Gevaert K., Van Breusegem F., Vertommen D., Young D., Rosado L.A., Messens J. (2018). Disulfide bond formation protects Arabidopsis thaliana glutathione transferase tau 23 from oxidative damage. Biochim. Biophys. Acta.

[64] Monroe J.D., Storm A.R. (2018). The Arabidopsis β-amylase (BAM) gene family: Diversity of form and function. Plant Sci..

[65] Dumont S., Bykova N.V., Khaou A., Besserour Y., Dorval M., Rivoal J. (2018). Arabidopsis thaliana alcohol dehydrogenase is differently affected by several redox modifications. PLoS One.

[66] Lamberti G., Drurey C., Soll J., Schwenkert S. (2011). The phosphorylation state of chloroplast transit peptides regulates preprotein import. Plant Signal. Behav..

[67] Holbrook K., Subramanian C., Chotewutmontri P., Reddick L.E., Wright S., Zhang H., Moncrief L., Bruce B.D. (2016). Functional analysis of semi-conserved transit peptide motifs and mechanistic implications in precursor targeting and recognition. Mol. Plant.

[68] de la Fuente van Bentem S., Anrather D., Dohnal I., Roitinger E., Csaszar E., Joore J., Buijnink J., Carreri A., Forzani C., Lorkovic Z.J. (2008). Site-specific phosphorylation profiling of Arabidopsis proteins by mass spectrometry and peptide chip analysis. J. Proteome Res..

[69] Nguyen-Kim H., San Clemente H., Balliau T., Zivy M., Dunand C., Albenne C., Jamet E. (2016). Arabidopsis thaliana root cell wall proteomics: Increasing the proteome coverage using a combinatorial peptide ligand library and description of unexpected Hyp in peroxidase amino acid sequences. Proteomics.

[70] Elmore J.M., Liu J., Smith B., Phinney B., Coaker G. (2012). Quantitative proteomics reveals dynamic changes in the plasma membrane during Arabidopsis immune signaling. Mol. Cell. Proteomics.

[71] Schwarzländer M., Dick T.P., Meyer A.J., Morgan B. (2016). Dissecting redox biology using fluorescent protein sensors. Antioxid. Redox Signal..

[72] Dietz K.-J. (2003). Redox control, redox signaling, and redox homeostasis in plant cells. Int. Rev. Cytol..

[73] Baier M., Dietz K.-J. (2005). Chloroplasts as source and target of cellular redox regulation: A discussion on chloroplast redox signals in the context of plant physiology. J. Exp. Bot..

[74] Kuźniak E., Kopczewski T. (2020). The chloroplast reactive oxygen species-redox system in plant immunity and disease. Front. Plant Sci..

[75] Lamb C., Dixon R.A. (1997). The oxidative burst in plant disease resistance. Annu. Rev. Plant Biol..

[76] Kiffin R., Christian C., Knecht E., Cuervo A.M. (2004). Activation of chaperone-mediated autophagy during oxidative stress. Mol. Biol. Cell..

[77] Salvemini D., Misko T.P., Masferrer J.L., Seibert K., Currie M.G., Needleman P. (1993). Nitric oxide activates cyclooxygenase enzymes. Proc. Natl. Acad. Sci. U. S. A..

[78] Rentel M.C., Lecourieux D., Ouaked F., Usher S.L., Petersen L., Okamoto H., Knight H., Peck S.C., Grierson C.S., Hirt H. (2004). OXI1 kinase is necessary for oxidative burst-mediated signalling in Arabidopsis. Nature.

[79] Baerenfaller K., Grossmann J., Grobei M.A., Hull R., Hirsch-Hoffmann M., Yalovsky S., Zimmermann P., Grossniklaus U., Gruissem W., Baginsky S. (2008). Genome-scale proteomics reveals Arabidopsis thaliana gene models and proteome dynamics. Science.

[80] Pérez-Ruiz J.M., Naranjo B., Ojeda V., Guinea M., Cejudo F.J. (2017). NTRC-dependent redox balance of 2-Cys peroxiredoxins is needed for optimal function of the photosynthetic apparatus. Proc. Natl. Acad. Sci. U. S. A..

[81] Mata-Pérez C., Spoel S.H. (2019). Thioredoxin-mediated redox signalling in plant immunity. Plant Sci..

[82] Hochmal A.K., Zinzius K., Charoenwattanasatien R., Gäbelein P., Mutoh R., Tanaka H., Schulze S., Liu G., Scholz M., Nordhues A., Offenborn J.N., Petroutsos D., Finazzi G., Fufezan C., Huang K. (2016). Calredoxin represents a novel type of calcium-dependent sensor-responder connected to redox regulation in the chloroplast. Nat. Commun..

[83] Chou I.T., Gasser C.S. (1997). Characterization of the cyclophilin gene family of Arabidopsis thaliana and phylogenetic analysis of known cyclophilin proteins. Plant Mol. Biol..

[84] Helm S., Baginsky S. (2018). MSE for label-free absolute protein quantification in complex proteomes. Plant Membrane Proteomics, 2018.

[85] Senkler J., Senkler M., Eubel H., Hildebrandt T., Lengwenus C., Schertl P., Schwarzländer M., Wagner S., Wittig I., Braun H. (2017). The mitochondrial complexome of Arabidopsis thaliana. Plant J..

[86] Li M., Ma X., Chiang Y.-H., Yadeta K.A., Ding P., Dong L., Zhao Y., Li X., Yu Y., Zhang L. (2014). Proline isomerization of the immune receptor-interacting protein RIN4 by a cyclophilin inhibits effector-triggered immunity in Arabidopsis. Cell Host Microbe.

[87] Marmagne A., Ferro M., Meinnel T., Bruley C., Kuhn L., Garin J., Barbier-Brygoo H., Ephritikhine G. (2007). A high content in lipid-modified peripheral proteins and integral receptor kinases features in the Arabidopsis plasma membrane proteome. Mol. Cell. Proteomics.

[88] Conrath U., Beckers G.J.M., Flors V., García-Agustín P., Jakab G., Mauch F., Newman M.-A., Pieterse C.M.J., Poinssot B., Pozo M.J. (2006). Priming: Getting ready for battle. Mol. Plant Microbe Interact..

[89] Goddard T.D., Huang C.C., Meng E.C., Pettersen E.F., Couch G.S., Morris J.H., Ferrin T.E. (2018). UCSF ChimeraX: Meeting modern challenges in visualization and analysis. Protein Sci..

[90] Pettersen E.F., Goddard T.D., Huang C.C., Couch G.S., Greenblatt D.M., Meng E.C., Ferrin T.E. (2004). UCSF Chimera—a visualization system for exploratory research and analysis. J. Comput. Chem..

[91] Navarathna C.M., Karunanayake A.G., Gunatilake S.R., Pittman C.U., Perez F., Mohan D., Mlsna T. (2019). Removal of Arsenic (III) from water using magnetite precipitated onto Douglas fir biochar. J. Environ. Manage..

[92] Akhtar A., Ghali L., Wang S.X., Bell C., Li D., Wen X. (2019). Optimisation of folate-mediated liposomal encapsulated arsenic trioxide for treating HPV-positive cervical cancer cells *in vitro*. Int. J. Mol. Sci..

[93] Olp M.D., Kalous K.S., Smith B.C. (2020). Icekat: An interactive online tool for calculating initial rates from continuous enzyme kinetic traces. BMC Bioinformatics.

[94] Lu W.-P., Fei L. (2003). A logarithmic approximation to initial rates of enzyme reactions. Anal. Biochem..

[95] Cornish-Bowden A. (1975). The use of the direct linear plot for determining initial velocities. Biochem. J..

[96] Lindsey B.E., Rivero L., Calhoun C.S., Grotewold E., Brkljacic J. (2017). Standardized method for high-throughput sterilization of Arabidopsis seeds. J. Vis. Exp..

[97] Bernsdorff F., Döring A.-C., Gruner K., Schuck S., Bräutigam A., Zeier J. (2016). Pipecolic acid orchestrates plant systemic acquired resistance and defense priming via salicylic acid-dependent and-independent pathways. Plant Cell..

[98] Schmittgen T.D., Livak K.J. (2008). Analyzing real-time PCR data by the comparative C T method. Nat. Protoc..

[99] Chambers M.C., MacLean B., Burke R., Amodei D., Ruderman D.L., Neumann S., Gatto L., Fischer B., Pratt B., Egertson J., Hoff K., Kessner D., Tasman N., Shulman N., Frewen B. (2012). A cross-platform toolkit for mass spectrometry and proteomics. Nat. Biotechnol..

[100] Käll L., Canterbury J.D., Weston J., Noble W.S., MacCoss M.J. (2007). Semi-supervised learning for peptide identification from shotgun proteomics datasets. Nat. Methods.

