# Supplementary material for: Arabidopsis thimet oligopeptidases are redox-sensitive enzymes active in the local and systemic plant immune response
Source: J Biol Chem. 2021 Apr 22;296:100695. doi: 10.1016/j.jbc.2021.100695 (PMC8215294; doi:10.1016/j.jbc.2021.100695)
Supplement: Figures S1 to S6 and Supplemental Methods [file mmc1.pdf]

Arabidopsis thimet oligopeptidases are redox-sensitive enzymes active in the local and systemic plant immune response

Authors

Thualfeqar Al-Mohanna [1], Najmeh Nejat [1], Anthony A. Iannetta [2], Leslie M. Hicks [2], George V. Popescu [3], and Sorina C. Popescu [1]*

Affiliation

[1] Department of Biochemistry, Molecular Biology, Entomology, and Plant Pathology, Mississippi State University, Mississippi State, MS, USA

[2] Department of Chemistry, The University of North Carolina at Chapel Hill, Chapel Hill, NC, USA

[3] Institute for Genomics, Biocomputing, and Biotechnology, Mississippi State University, Mississippi State, MS, USA

*Corresponding author

Supporting information

List of the materials included:

I. Supporting figures and legends
II. Supporting Methods

I. Supporting Figures

Protein	Residue	Buried Coeff.
TOP1	Cys 29	0%
	Cys 42	48%
	Cys 52	0%
	Cys 548	100%
	Cys 611	100%
	Cys 699	78%
$^{\Delta SP}$TOP1	Cys 548	100%
	Cys 611	100%
	Cys 699	77%
TOP2	Cys 405	40%
	Cys 460	100%
	Cys 523	100%
	Cys 611	85%

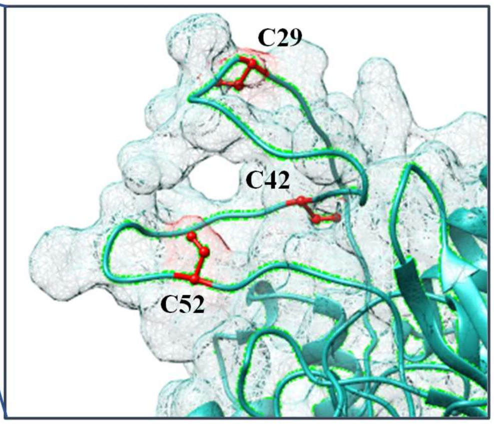

SFig 1 Structural and physicochemical characteristics of TOP1 and TOP2. A) A Chimera-derived 3-dimensional (3D) model of full-length TOP1, showing the signal peptide and the three cysteine residues' position, shown using Ribbon-rounded and surface-mesh with 90% transparency for TOP1 structure; cysteines are shown in the ball-and-stick representation; percentages of calculated solvent-exposure for each Cys are shown. C) Thermal denaturation of TOP1 and TOP2 assessed by measuring the intrinsic fluorescence of purified proteins during incremental increase (20-65 ^{o}C, green data points) and decrease (65 to 20 ^{o}C, blue data points) in temperature at Ex_{280nm} and $Em_{(300\text{-}500)\ nm}$; the graphs show the linear regression fit of TOP1 and TOP2 under denaturing and renaturing conditions at Ex_{280nm} and Em_{338nm}.

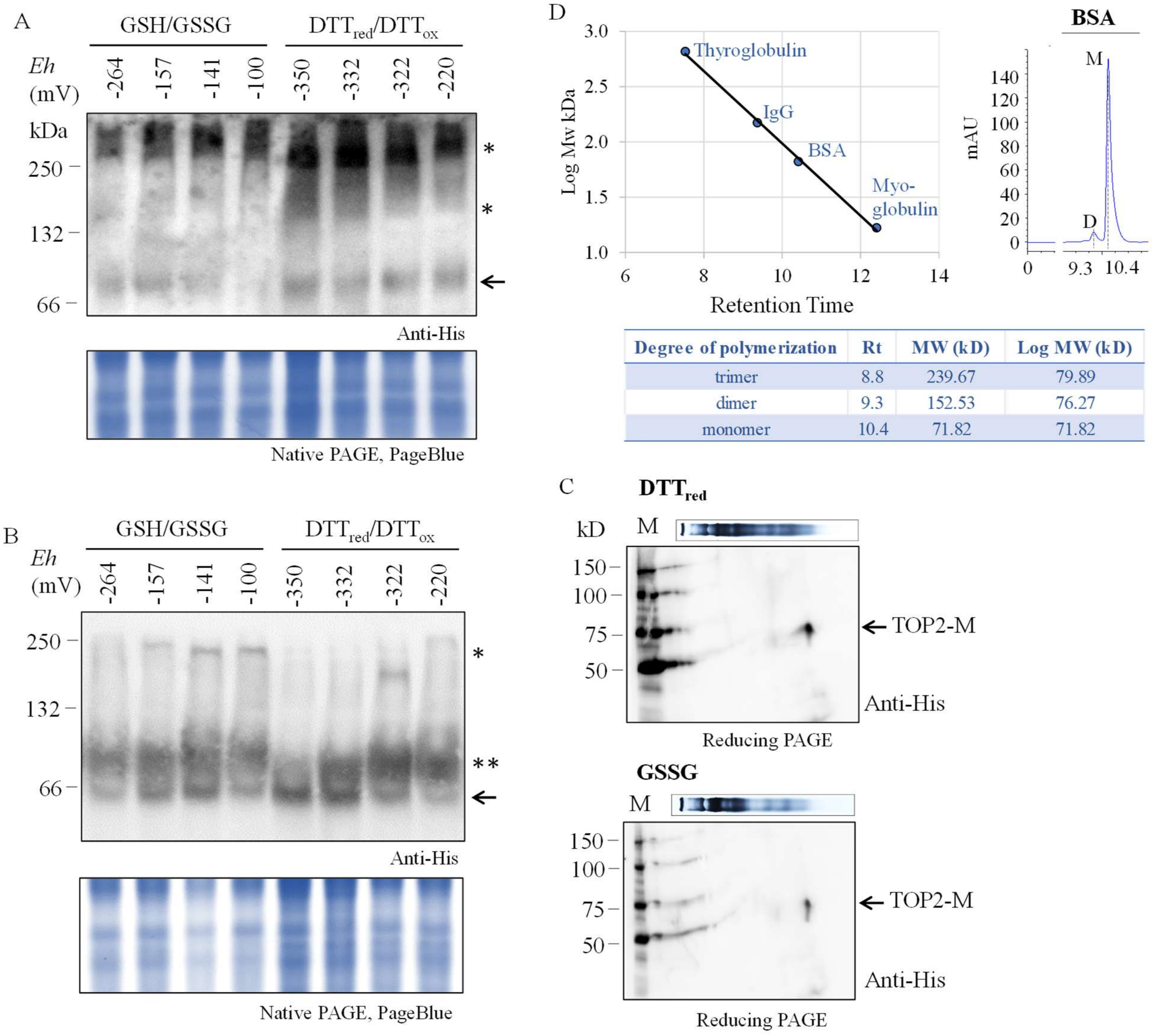

Degree of polymerization	Rt	MW (kD)	Log MW (kD)
trimer	8.8	239.67	79.89
dimer	9.3	152.53	76.27
monomer	10.4	71.82	71.82

SFig 2 A and B) Immunoblots of total extracts from *E. coli* cultures expressing TOP1-His (A) or TOP2-His (B) subjected to redox treatments, run on native PAGE and probed with anti-His antibodies; arrows show the position of monomers and asterisk (* and **) the position of higher-MW bands. Page blue staining show equal loading. The redox couples used for treatments (DTT_{red}-$DTTo_{x}$ and GSH-GSSG) and the redox potentials obtained by mixing the redox reagents (*Eh*) are listed above each lane. C) TOP2 monomers (M) detected using 2D electrophoretic separation of total extracts from *E. coli* cells treated with DTT_{red} or GSSG; total protein was separated on native PAGE to obtain the gel strips which were then loaded into reducing PAGE for separation in a 2nd dimension, followed by immunoblotting with anti-His antibodies. Molecular weight markers (M) are shown. D) Regression curve for mass calculation produced using protein markers eluted through the SEC-HPLC column. Bovine serum albumin (BSA), shown on the right, elutes as a dimer (D) and monomer (M), with peak retention times corresponding to their respective molecular weights. The Table shows the MW and Rt of TOP1 monomer, dimer, and trimer.

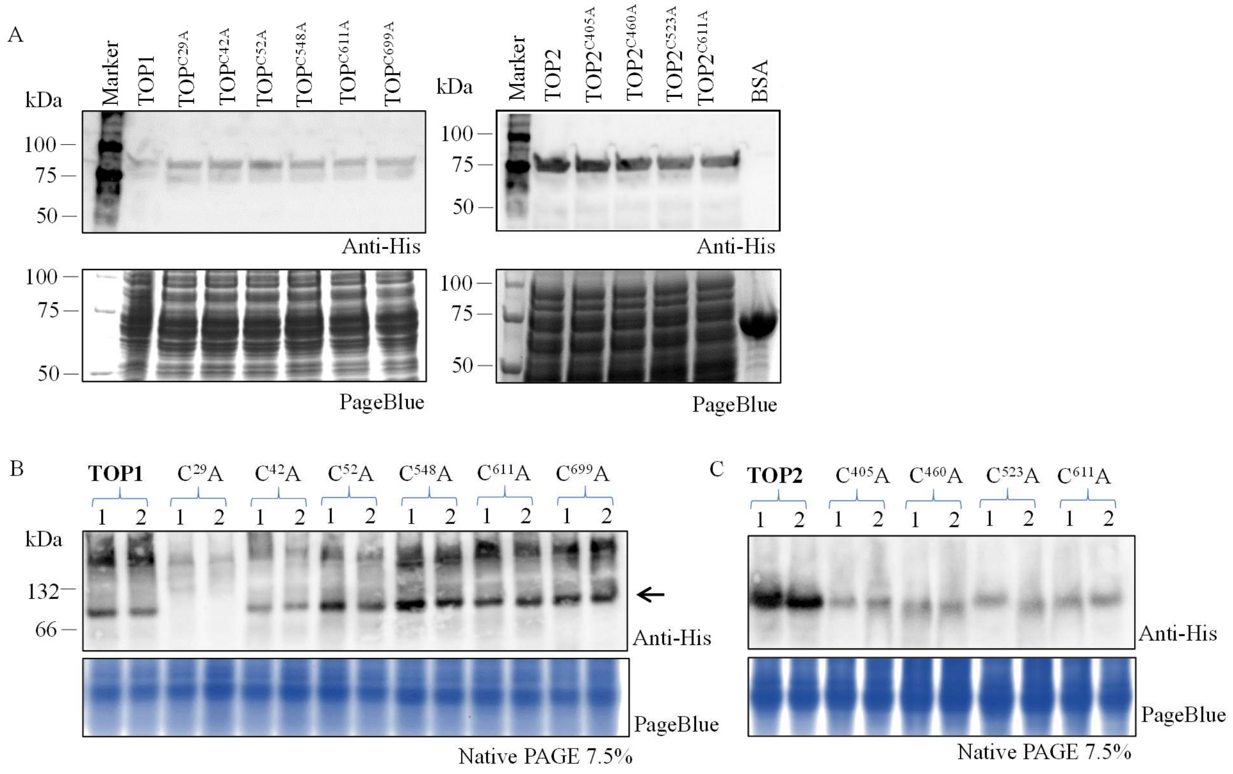

SFig 3 A) Representative immunoblots of total protein extracts from *E. coli* cells expressing *TOP1* and *TOP2* native and mutant isoforms**.** The blots were probed with anti-His antibodies or stained with PageBlue stain to show equal loading. B) Purified recombinant TOP1 native and mutant proteins treated with GSH (1) and GSSG (2). Arrow shows position of monomers. C) Purified recombinant TOP2 native and mutant proteins treated with GSH (1) and GSSG (2). In B and C, proteins were run on native PAGE, blotted, and probed with anti-His antibody or stained with PageBlue. The molecular weight markers are shown on the left of each blot.

TOP1 (AT5G65620)

Matched peptides shown in **bold red**

Monomeric

Protein sequence coverage: 34%

```
  1 MLMATPTSRA SLNLLRRSPK PKYFSSSSCH FRPSTFRKSY PCPIWSSSFS
 51 FCLPPPRSTT STSLSSSSFR PFSSPPSMSS AAAAAVESVV SDETLSSNPL
101 LQDFDFPPFD SVDASHVRPG IRALLQHLEA ELEELEKSVE PTWPKLVEPL
151 EKIVDRLTVV WGMINHLKAV KDTPELRAAI EDVQPEKVKF QLRLGQSKPI
201 YNAFKAIRES PDWSSLSEAR QRLVEAQIKE AVLIGIALDD EKREEFNKIE
251 QELEKLSHKF SENVLDATKK FEKLITDKKE IEGLPPSALG LFAQAAVSKG
301 HENATAENGP WIITLDAPSY LPVMQHAKNR ALREEVYRAY LSRASSGDLD
351 NTAIIDQILK LRLEKAKLLG YNNYAEVSMA MKMATVEKAA ELLEKLRSAS
401 WDAAVQDMED LKSFAKNQGA AESDSMTHWD TTFWSERLRE SKYDINEEEL
451 RPYFSLPKVM DGLFSLAKTL FGIDIEPADG LAPVWNNDVR FYRVKDSSGN
501 PIAYFYFDPY SRPSEKRGGA WMDEVVSRSR VMAQKGSSVR LPVAHMVCNQ
551 TPPVGDKPSL MTFREVETVF HEFGHALQHM LTKQDEGLVA GIRNIEWDAV
601 ELPSQFMENW CYHRDTLMSI AKHYETGETL PEEVYKKLLA ARTFRAGSFS
651 LRQLKFASVD LELHTKYVPG GPESIYDVDQ RVSVKTQVIP PLPEDRFLCS
701 FSHIFAGGYA AGYYSYKWAE VLSADAFSAF EDAGLDDIKA VKETGQRFRN
751 TILALGGGKA PLKVFVEFRG REPSPEPLLR HNGLLAASAS A
```

Dimeric

Protein sequence coverage: 49%

```
  1 MLMATPTSRA SLNLLRRSPK PKYFSSSSCH FRPSTFRKSY PCPIWSSSFS
 51 FCLPPPRSTT STSLSSSSFR PFSSPPSMSS AAAAAVESVV SDETLSSNPL
101 LQDFDFPPFD SVDASHVRPG IRALLQHLEA ELEELEKSVE PTWPKLVEPL
151 EKIVDRLTVV WGMINHLKAV KDTPELRAAI EDVQPEKVKF QLRLGQSKPI
201 YNAFKAIRES PDWSSLSEAR QRLVEAQIKE AVLIGIALDD EKREEFNKIE
251 QELEKLSHKF SENVLDATKK FEKLITDKKE IEGLPPSALG LFAQAAVSKG
301 HENATAENGP WIITLDAPSY LPVMQHAKNR ALREEVYRAY LSRASSGDLD
351 NTAIIDQILK LRLEKAKLLG YNNYAEVSMA MKMATVEKAA ELLEKLRSAS
401 WDAAVQDMED LKSFAKNQGA AESDSMTHWD TTFWSERLRE SKYDINEEEL
451 RPYFSLPKVM DGLFSLAKTL FGIDIEPADG LAPVWNNDVR FYRVKDSSGN
501 PIAYFYFDPY SRPSEKRGGA WMDEVVSRSR VMAQKGSSVR LPVAHMVCNQ
551 TPPVGDKPSL MTFREVETVF HEFGHALQHM LTKQDEGLVA GIRNIEWDAV
601 ELPSQFMENW CYHRDTLMSI AKHYETGETL PEEVYKKLLA ARTFRAGSFS
651 LRQLKFASVD LELHTKYVPG GPESIYDVDQ RVSVKTQVIP PLPEDRFLCS
701 FSHIFAGGYA AGYYSYKWAE VLSADAFSAF EDAGLDDIKA VKETGQRFRN
751 TILALGGGKA PLKVFVEFRG REPSPEPLLR HNGLLAASAS A
```

SFig 4 Confirmation of TOP1 in trypsin and chymotrypsin in-gel digested protein bands. Identified peptides with Mascot peptide score > 13 in protein bands excised at mass ranges corresponding to monomeric and dimeric TOP1.

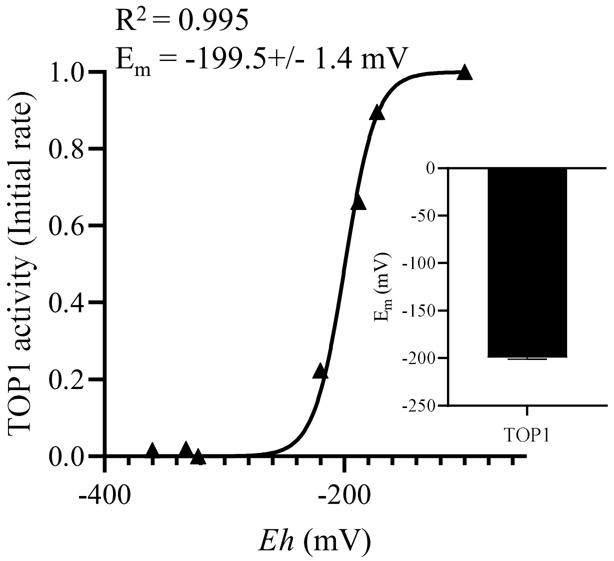

SFig 5 Purified recombinant TOP1 was incubated in DTTred/DTTox or GSH/GSSG solutions at defined thiol/disulfide ratios. The triangles show the oxidation-reduction titration of TOP1, and the solid line shows the fit of the data to a two-electron Nernst curve. The summary of the analysis is shown in the inset. The R^2 shows the goodness of fit. Data were acquired in two independent experiments; one representative is shown.

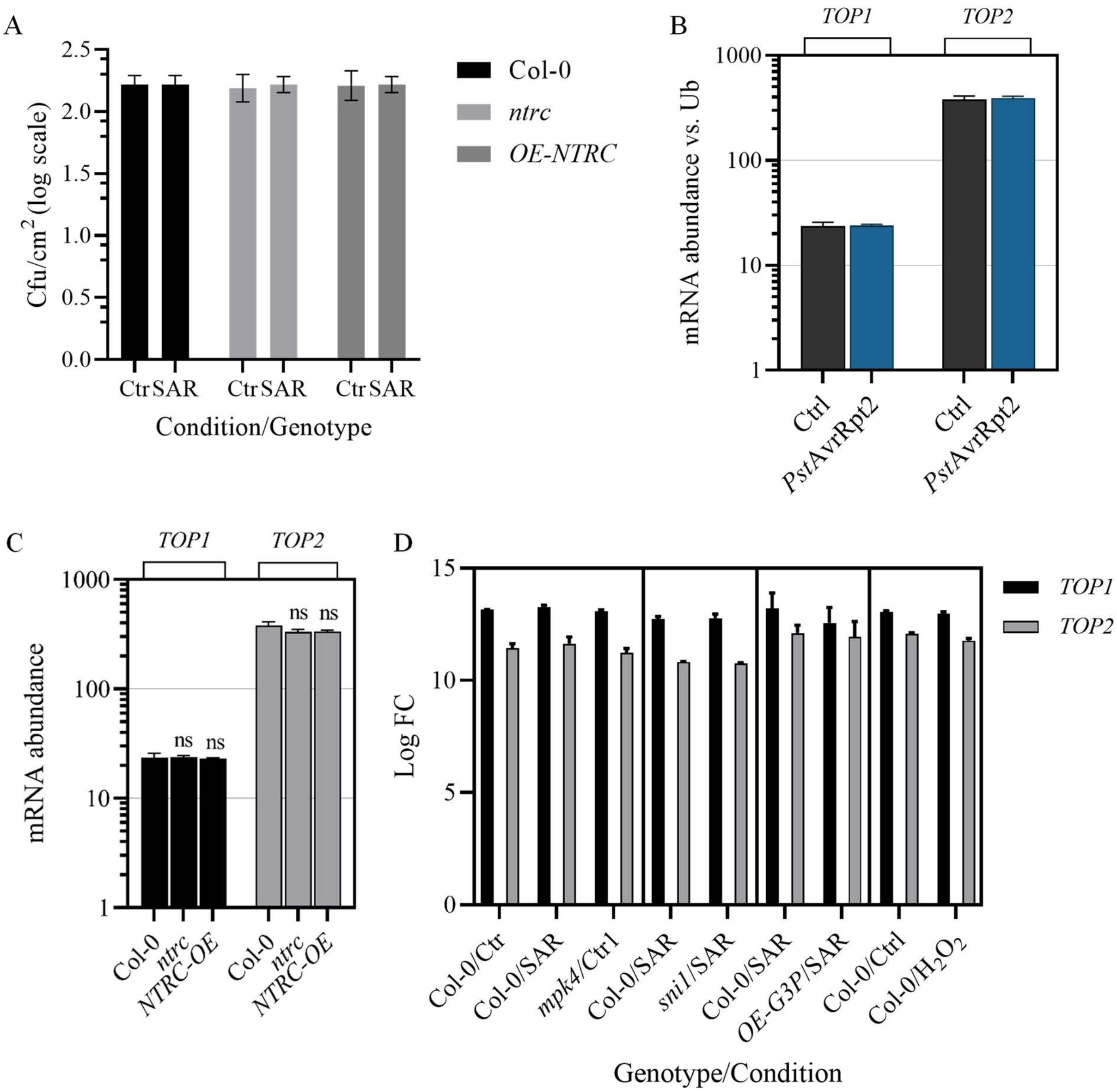

SFig 6 A) Quantification of *P. syringae* DC3000 growth (Colony-forming units, Cfu/cm^2) in plants treated as shown in Fig 6, at 0 hours post-inoculation; n=4. B and C) *TOP1* and *TOP2* mRNA abundance in Col-0 wild type buffer-infiltrated (Ctrl) and *P. syringae* AvrRpt2-infiltrated plants (B), and in *ntrc* and *OE-NTRC* mutant lines; TOP transcript abundance was normalized against a ubiquitin housekeeping gene (n=3). D) Systemic acquired immune response (SAR) and H_2O_2 treatment have a negligible impact on the expression level of *TOP1* and *TOP2* in Col-0, immune-defective mutant (*sni1*), and redox mutant (*OE-G3P*) lines.

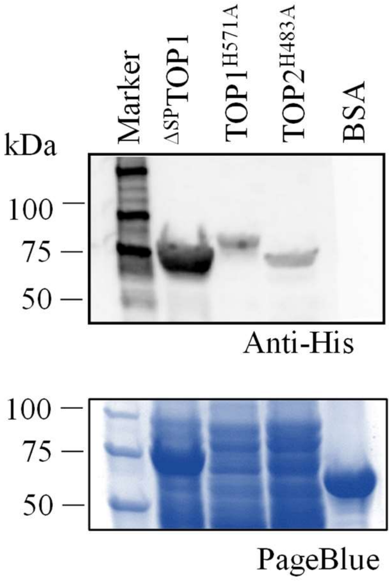

SFig 7 Immunoblots of total protein extracts from *E. coli* cells expressing TOP native and mutant isoforms. The blots were probed with anti-His antibodies. PageBlue staining shows equal loading.

II. Supplementary Methods

Cloning and site-directed mutagenesis to generate TOP cysteine mutants

TOP1 and TOP2 cDNAs were cloned into pET-28a Agilent protein expression vector in translational frame with 6x His N-terminal tags. Cysteine mutagenesis was performed by site-directed mutagenesis, using the wild type cDNAs, gene-specific primers and Phusion High-Fidelity DNA Polymerase (New England Biolabs).

TOP1 primers

TOP1 Forward Primer (T1F) 5`-ATATGCTAGCTTAATGGCGACTCCAACGTC-3`

TOP1 Reverse Primer (T1R) 5`-ATATGTCGACTTAAGCAGAAGCAGAGGCAG-3`

^{SP}TOP1F Forward Primer 5`-ATATGCTAGCTCCGACGAGACTCTTTCCTC-3

*TOP*1^{C29A} Forward Primer (T1^{C29A}F) 5`-TCATCTTCTTCTGCACATTTTCGTCCC-3`

*TOP*1^{C42A} Forward Primer (T1^{C42A}F) 5`-AATCATATCCTGCACCCATCTGGTCTTC-3`

*TOP*1^{C52A} Forward Primer (T1^{C52A}F) 5`-TCTTTCTCTTTTGCACTCCCTCCTCC-3`

*TOP*1^{C548A} Forward Primer (T1^{C548A}F) 5`-ACACATGGTCGCAAACCAAACTCC-3`

*TOP*1^{C611A} Forward Primer (T1^{C611A}F) 5`-GGAGAATTGGGCATACCACAGGG-3`

*TOP*1^{C699A} Forward Primer (T1^{C699A}F) 5`-AGGATAGATTTCTCGCAAGCTTCAGTCAC-3`

*TOP*1^{C29A} Reverse Primer (T1^{C29A}R) 5`-GGGACGAAAATGTGCAGAAGAAGATG-3`

*TOP*1^{C42A} Reverse Primer (T1^{C42A}R) 5`-ACCAGATGGGTGCAGGATATGATTTACG-3`

*TOP*1^{C52A} Reverse Primer (T1^{C52A}R) 5`-GGAGGAGGGAGTGCAAAAGAGAAAGAAG-3`

*TOP*1^{C548A} Reverse Primer (T1^{C548A}R) 5`-GAGTTTGGTTTGCGACCATGTGTG-3`

*TOP*1^{C611A} Reverse Primer (T1^{C611A}R) 5`-TCCCTGTGGTATGCCCAATTCTCC-3`

*TOP*1^{C699A} Reverse Primer (T1^{C699A}R) 5`-TGTGACTGAAGCTTGCGAGAAATCTATCC-3`

TOP1^{H571A} Forward Primer (T1^{H571A}F) 5`-GAGACAGTATTTGCTGAATTTGGACATGCTC-3`

TOP1^{H571A} Reverse Primer (T1^{H571A}R) 5`- GAGCATGTCCAAATTCAGCAAATACTGTCTC-3`

TOP2 primers

TOP2 Forward Primer (T2F) 5`-ATATGCTAGCGCTTCTGAAGATACTCTCTCCTC-3`

TOP2 Reverse Primer (T2R) 5`-ATATGTCGACTCAAGCAGAAGCAGCCAAG-3`

*TOP*2^{C405A} Forward Primer (T2^{C405A}F) 5`-
CGATGTTAGGTTCTACGCAGTCAAAGATTCTTC-3`

*TOP*2^{C460A} Forward Primer (T2^{C460A}F) 5`-CTCAAATGGTCGCAAACCAAACTCC-3`

*TOP*2^{C523A} Forward Primer (T2^{C523A}F) 5`-TATGGAGAACTGGGCATACCACAGGG-3`

*TOP*2^{C611A} Forward Primer (T2^{C611A}F) 5`-CTGAAGATAGATTCCTCGCAAGCTTCAGTC-3`

*TOP*2^{C405A} Reverse Primer (T2^{C405A}R) 5`-AGAATCTTTGACTGCGTAGAACCTAACATCG-3`

*TOP*2^{C460A} Reverse Primer (T2^{C460A}R) 5`-GAGTTTGGTTTGCGACCATTTGAGC-3`

*TOP*2^{C523A} Reverse Primer (T2^{C523A}R) 5`-ATCCCTGTGGTATGCCCAGTTCTCC-3`

*TOP*2^{C611A} Reverse Primer (T2^{C611A}R) 5`-TGACTGAAGCTTGCGAGGAATCTATCTTC-3`

TOP2^{H483A} Forward Primer (T2^{H483A}F) 5`-GTAGAGACTGTGTTCGCTGAATTTGGCCAT-3`

TOP2^{H483A} Reverse Primer (T2^{H483A}R) 5`-CATGGCCAAATTCAGCGAACACAGTCTCTA-3`

The mutagenesis was performed as follows. In the first PCR run (30 cycles) using a Phusion High-Fidelity DNA Polymerase, primers pairs were used to obtain two overlapping amplicons for each TOP1 and TOP2 mutants. One amplicon included the 5' fragment up to the mutagenesis site and another amplicon included the 3' fragment up to the mutagenesis site. The amplicons were separated in agarose gels (0.8%), and the bands were collected and purified by using the QIAquick gel extraction Kit. To obtain the full-length mutagenized cDNAs, the overlapping amplicons were mixed, annealed, and amplified using forward and reverse primers. The final PCR products were separated on agarose gels (0.8%), purified using the QIAquick gel extraction Kit (Qiagen), and cloned into pET-28a using Nhe I and Sal I restriction enzymes (NEB) and T4 DNA ligase (NEB), transformed into NEB 10-beta *E. coli* competent cells (High Efficiency) and plated on LB plates with Kan (50mg/L). Positive colonies were selected by colony PCR, plasmids purified and sequenced (https://www.eurofinsgenomics.com) to confirm the mutation. Positive and verified plasmids were transformed into transferred to NiCo21 (DE3) *E. coli* (C2529H) competent cells (NEB) and used for protein expression and purification.

Redox titrations

All solutions were prepared in 50mM Tris-buffer pH 7.0. All calculations were done using the Nernst equation by taking the potentials of redox couples calculated in (Zannini, Couturier, Keech & Rouhier 2017).

Zannini F., Couturier J., Keech O. & Rouhier N. (2017) In Vitro Alkylation Methods for Assessing the Protein Redox State. In *Photorespiration*. pp. 51–64. Springer.
